# Plant-Derived Functional Molecules for Photoelectrochemical Water Splitting: From Molecular Design to Hybrid Materials Engineering

**DOI:** 10.3390/ijms27209326

**Published:** 2026-10-20

**Authors:** Sangmin Kim, Inseo Hwang, Gihoon Kim, Hyeonseok Yoon

**Affiliations:** 1 Department of Polymer Engineering, Graduate School, Chonnam National University, 77 Yongbong-ro, Gwangju 61186, Republic of Korea; 2 School of Polymer Science and Engineering, Chonnam National University, 77 Yongbong-ro, Gwangju 61186, Republic of Korea

**Keywords:** photoelectrochemical water splitting, plant-derived molecules, metal–phenolic networks, interfacial charge transfer, BiVO_4_ photoanodes, molecular interface engineering, photoelectrodes

## Abstract

Plant-derived molecules offer tunable coordination and surface chemistry for photoelectrochemical (PEC) water splitting. A Scopus survey retrieved 45 documents linking both topics in 2005–2025, equivalent to 0.34% of the PEC literature. A supplementary search added 15 records, and screening retained 26 research articles. The review organizes molecular functions into light harvesting, charge-transfer mediation, coordination of catalytic overlayers, and passivation. A molecular readiness scale and a validation protocol connect molecular identity to verified device performance. Hybrid layers combining plant ligands with Co, Ni, or Fe raised BiVO_4_ photocurrents to approximately 3–6 mA cm^−2^ at 1.23 V vs. the reversible hydrogen electrode (RHE). Comparable inorganic overlayers preclude a general claim of superior activity. Ambient solution processing and control of interfacial coordination provide the principal design opportunities. Two-electron phenol/quinone potentials do not establish the feasibility of one-electron hole relay, which requires independent kinetic and redox evidence. At a coverage of 0.1 nmol cm^−2^, a chlorophyll layer has a calculated photocurrent upper bound of approximately 0.05 mA cm^−2^ within 640–680 nm alone. Broadband enhancement requires wavelength-resolved attribution. Operation for 1000 h at 1 mA cm^−2^ and a loading of 1 nmol cm^−2^ corresponds to approximately 3.7 × 10^7^ electrons per initially loaded molecule. The proposed framework links molecular structure, interfacial function, and product verification to guide durable molecular photoelectrodes.

## 1. Introduction

Hydrogen is a core feedstock for oil refining and for ammonia and methanol synthesis, and global demand reached almost 100 Mt in 2024 [1]. Unabated fossil fuels supplied nearly all of the demand, whereas low-emissions production was projected to reach only about 1 Mt in 2025, below 1% of the total [1]. Decarbonizing the hydrogen supply therefore requires routes that store renewable energy directly in chemical bonds. Photoelectrochemical (PEC) water splitting satisfies the requirement by integrating light absorption, charge separation, and catalysis within a single semiconductor/electrolyte assembly [2,3]. Such integration removes the separate photovoltaic and electrolyzer units of indirect systems and gives a compact route to solar hydrogen.

More than five decades after the pioneering demonstration on TiO_2_ by Fujishima and Honda [2], the efficiency and durability of PEC devices still fall short of practical targets [4]. The dominant losses arise at the semiconductor/electrolyte junction. Wide-bandgap oxides, including TiO_2_ and ZnO, absorb only ultraviolet light. Visible-light absorbers such as BiVO_4_ and α-Fe_2_O_3_ suffer from surface recombination, sluggish four-electron oxygen evolution, and photocorrosion [5,6]. Severe surface recombination, for instance, keeps the photocurrent of bare BiVO_4_ far below its theoretical limit of approximately 7.5 mA cm^−2^ [7]. Elemental doping, heterojunction formation, cocatalyst loading, and protective overlayers have each mitigated specific losses [6,8]. Many of the resulting electrodes, however, rely on noble or critical metals, vacuum deposition, or high-temperature processing, which erode the cost and environmental advantages of solar hydrogen.

Molecular engineering of the photoelectrode surface provides a complementary strategy. Organic molecules can adjust light absorption, energy-level alignment, charge extraction, and surface chemistry with sub-nanometer precision under ambient processing. Dye-sensitized photoelectrochemical cells have demonstrated the value of molecular components [9], although many of the best-performing assemblies employ Ru-based chromophores and catalysts obtained through multistep synthesis.

Oxygenic photosynthesis has already solved several of the problems a photoelectrode faces. Chlorophylls absorb intensely in the blue and the red; carotenoids cover part of the blue–green gap left between them, and also quench chlorophyll triplets and singlet oxygen. Quinones carry electrons within photosystem II (PSII). The coordination chemistry belongs to the polyphenols, which chelate metal ions and scavenge radicals, while betalains contribute visible absorption together with carboxyl groups that bind oxide surfaces. Light harvesting, charge relay, catalytic site assembly, and protection against oxidative damage are the same four tasks a molecule must perform at a semiconductor interface. Herein, plant-derived functional molecules denote metabolites and pigments whose native scaffolds are biosynthesized by plants, together with semi-synthetic derivatives that retain the native chromophore or ligand framework. Agricultural and food-processing residues can supply many of them. Plant biomass reaches energy materials by either of two routes. Carbonization converts lignocellulosic residues into porous carbons and metal–carbon hybrids whose properties follow from precursor chemistry, morphology, and heat treatment instead of from any single constituent [10,11,12]. Molecular utilization, the subject of this review, preserves the native chromophore or ligand and places it at a semiconductor interface.

Existing reviews have treated natural pigments mainly as sensitizers for TiO_2_ photoanodes [13] or have surveyed photosensitizers of all origins with an emphasis on toxicity ranking [14]. Reviews of dye-sensitized PEC cells center on chromophore–catalyst assemblies [9], and semi-artificial photosynthesis relies on intact photosystems and enzymes rather than small molecules [15]. Reviews of BiVO_4_ photoanodes survey doping, heterojunction formation, and inorganic overlayers without treating plant-derived molecules as interfacial functional components [8]. Recent studies have revealed functions of plant molecules that extend well beyond sensitization. Tannic acid–metal coordination layers on BiVO_4_ served simultaneously as hole-transfer mediators and cocatalysts [7,16]. A Co-based layer, for example, generated Co^3+^ active sites and raised the photocurrent at 1.23 V vs. the reversible hydrogen electrode (RHE) approximately threefold [7]. Conformal tannic acid–NiFe networks accelerated water oxidation and protected Mo-doped BiVO_4_ from photocorrosion [17]. Organometallic overlayers derived from natural molecules likewise delivered high-efficiency PEC water splitting [18]. Caffeic acid and flavonoid ligands acted as molecular bridges and electronic modulators between BiVO_4_ and Ni-based cocatalysts [19,20]. To the best of our knowledge, no review has organized such multifunctional roles into a structure–function framework.

**Figure 1:**
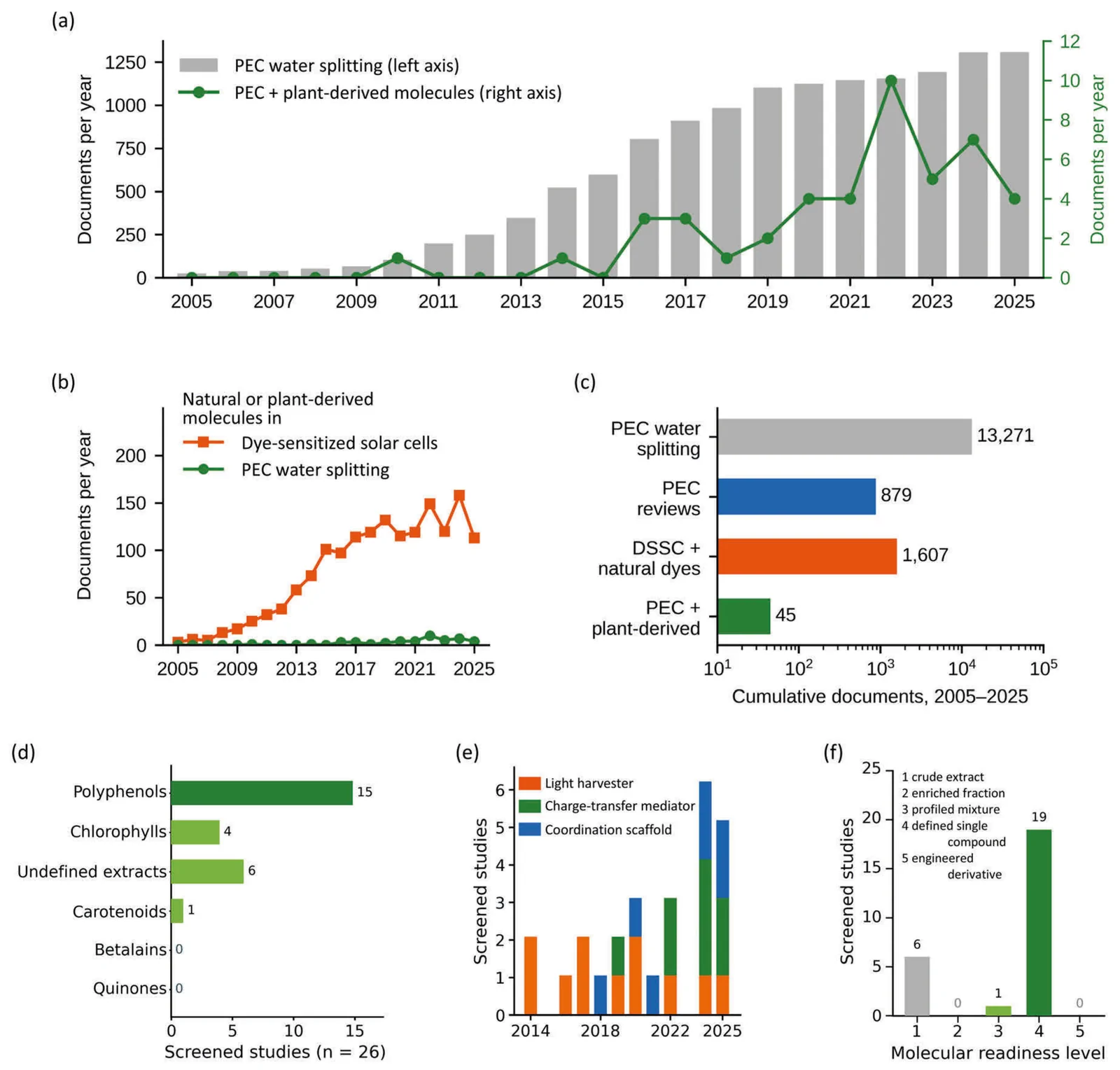
Scopus bibliometric analysis for 2005–2025. (**a**) Annual documents on PEC water splitting and the subset mentioning plant-derived molecules. (**b**) Annual documents on natural or plant-derived molecules in DSSCs and PEC water splitting. (**c**) Cumulative query counts on a logarithmic scale. (**d**) Molecular classes in the 26 retained research articles. (**e**) Annual distribution by assigned primary role: light harvester (orange), charge-transfer mediator (green), and coordination scaffold (blue). Each study is counted once, and secondary functions are not counted separately; no retained study was assigned passivation alone as its primary role. (**f**) Molecular readiness levels (MRLs), assigned from the reported identity of the molecular component. Query strings and screening decisions appear in Tables S1–S4. For MRL 3, the mixture profile denotes reported constituent identities, not quantitative analytical profiling.

A bibliometric survey of the Scopus database quantifies the gap (Figure 1). Title, abstract, and keyword searches retrieved 13,271 documents on PEC water splitting published between 2005 and 2025, including 879 reviews (search date: 21 September 2026; query strings in Table S1). Only 45 of these documents also mentioned plant-derived molecules, corresponding to 0.34% of the PEC literature. Natural dyes in dye-sensitized solar cells (DSSCs), by comparison, accumulated 1607 documents over the same period, roughly 36 times as many. Plant chemistry has thus been explored extensively for photovoltaics and only marginally for solar fuels. The PEC subset has nevertheless grown recently, with 43 of the 45 documents of the primary query published after 2015 and an annual maximum of 10 documents in 2022.

A supplementary search using terms omitted from the primary query returned 15 additional records, giving 60 records for screening. Screening retained 26 research articles and one review (Table S2). The 33 excluded records involved synthesis reagents, proteins or intact tissue, non-plant modifiers, carbonized precursors, or applications outside the scope. The retained articles comprised 15 polyphenol studies, four chlorophyll studies, six studies of compositionally undefined extracts, and one carotenoid study (Figure 1d). No retained study examined purified betalains or plant quinones. The retained review treated natural dyes as TiO_2_ sensitizers [13]. Keyword retrieval remains sensitive to terminology, so the counts describe the specified search rather than the complete literature.

The literature also shares three methodological weaknesses. First, many studies employ crude extracts of undefined composition, which obscures structure–property relationships and limits reproducibility. Second, polyphenols and quinones oxidize readily, so photocurrent reported without Faradaic verification may partly reflect molecular self-oxidation rather than water oxidation. Third, sustainability is often inferred from natural origin alone, without evaluation of extraction burdens, batch variability, or device lifetime.

**Figure 2:**
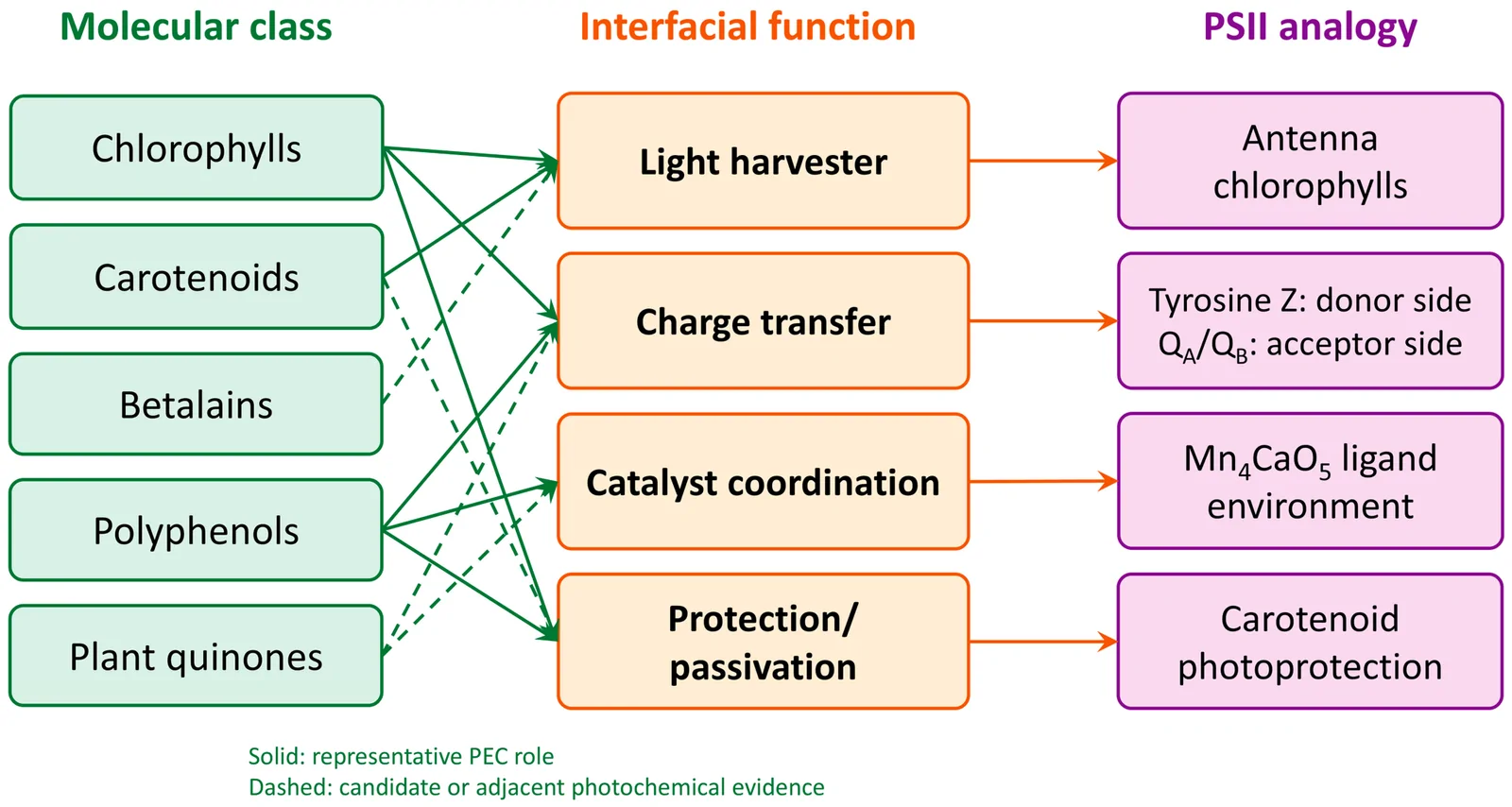
Functional framework for plant-derived molecules at PEC interfaces. Solid connections indicate representative roles discussed for molecular PEC electrodes; dashed connections identify candidate roles or roles supported by adjacent photochemical evidence. The PSII column provides functional analogies, not structural equivalence. Chlorophyll derivatives can also mediate hole transport and protect the host. Photoprotection and electronic surface passivation require different mechanistic tests.

Here we address the conceptual gap together with the methodological weaknesses outlined above. The conceptual gap is the absence of a framework that links molecular structure to interfacial function, which leaves multifunctional behavior reported case by case and blocks comparison across semiconductor hosts. The methodological gaps concern what a reported photocurrent establishes: the composition of the molecular layer, the origin of the measured current, and the evidence behind claims of sustainability. Plant-derived molecules are classified according to four interfacial roles: light harvester, charge-transfer mediator, coordination scaffold for catalytic overlayers, and protective passivator (Figure 2).

The bibliometric corpus covers 2005–2025 and includes studies in which a plant-derived molecule or extract remains a functional component of a photoanode or photocathode during PEC water splitting. Structural definition is graded rather than required for inclusion. Carbonized precursors, extracts consumed during synthesis, and systems restricted to other reactions are excluded from the corpus [21,22]. The supporting literature on photocatalytic suspensions, DSSCs, and PSII informs molecular properties and mechanisms without entering the screening statistics. The mechanistic discussion and representative performance compilation additionally include the 2026 quercetin–NiCo study [20]. That study falls outside the bibliometric window and is not counted in Figure 1 or Table S3. Differences in host, electrolyte, and operating conditions limit comparisons across the performance compilation.

Section 2 describes five molecular classes and their intrinsic properties, and Section 3 derives design principles for energy alignment, anchoring, stability, and data-driven identification. Section 4 examines hybridization strategies according to functional role and semiconductor host. Section 5 proposes a protocol for mechanistic validation and quantitative benchmarking, and Section 6 evaluates sustainability beyond natural origin. Section 7 outlines future directions, including photosynthesis-inspired multifunctional assemblies. Linking molecular origin, interfacial function, and verified device metrics is what turns plant-derived molecules from inexpensive substitutes for synthetic dyes into rationally engineered components of solar fuel devices.

## 2. Plant-Derived Functional Molecules: Structural Classes and Intrinsic Properties

**Figure 3:**
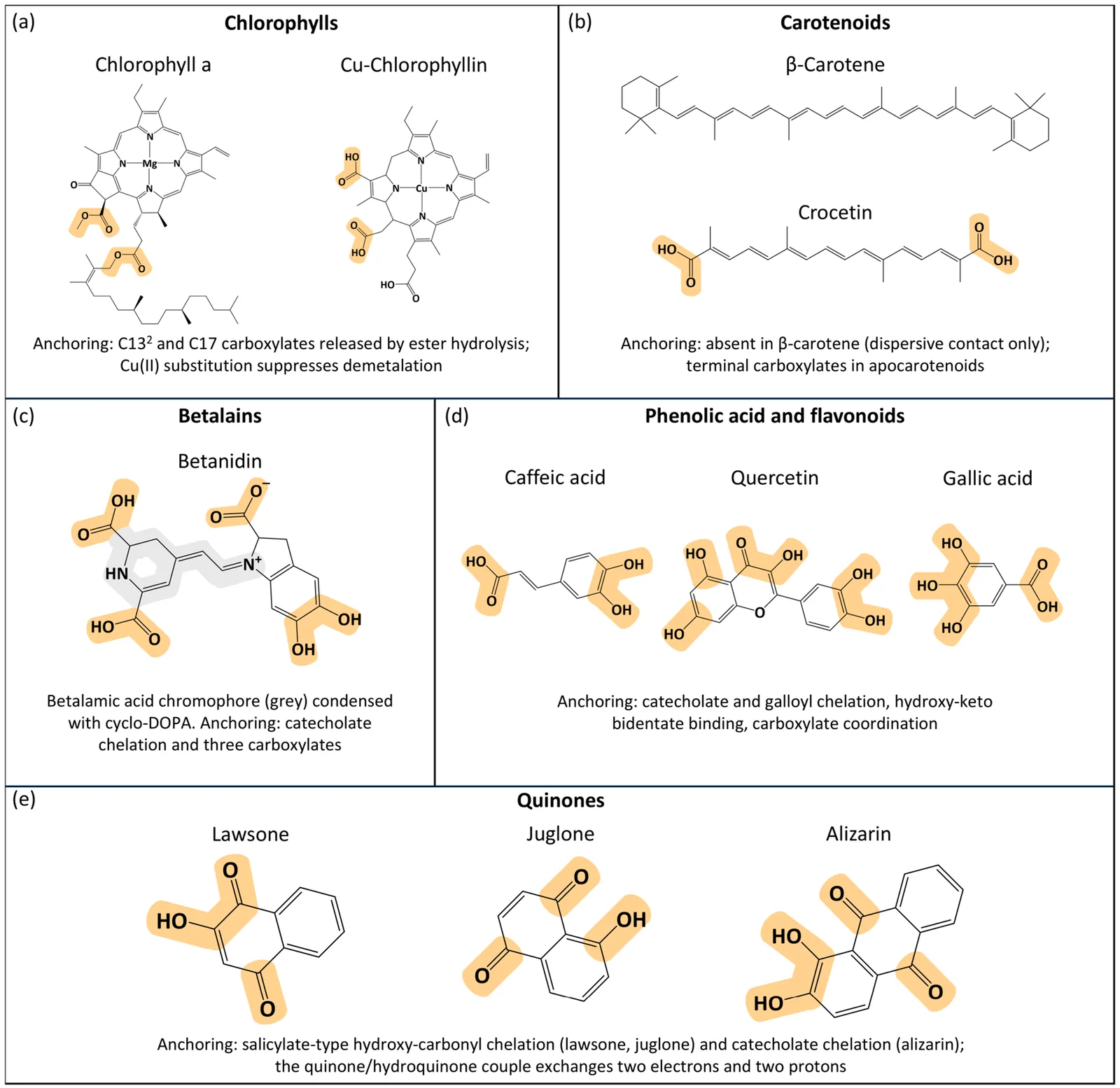
Representative structures of the five classes of plant-derived molecules, annotated with the motif that governs attachment to a semiconductor surface. (**a**) Chlorophyll a carries a C13^2^ methoxycarbonyl group and a C17 phytyl ester, and both require hydrolysis before carboxylate anchoring becomes available. Cu-chlorophyllin, the saponified derivative, exposes free carboxylates and resists demetalation through Cu(II) substitution. (**b**) β-Carotene combines eleven conjugated double bonds with a pure hydrocarbon skeleton and offers no anchoring group. Crocetin, an acyclic C_20_ apocarotenoid, terminates its seven conjugated double bonds in two carboxylic acids. (**c**) Betanidin arises from condensation of betalamic acid (shaded) with cyclo-DOPA, which places one catechol and three carboxylates on a single chromophore. Glycosylation at C5 yields betanin and blocks one catechol oxygen. (**d**) Caffeic acid, quercetin, and gallic acid, the galloyl unit of tannic acid; catecholate, galloyl, and hydroxy–keto motifs chelate surface metal ions, while carboxylates provide additional binding. (**e**) Lawsone and juglone chelate through a salicylate-type hydroxy–carbonyl pair, whereas alizarin binds through a 1,2-dihydroxy catecholate. The quinone/hydroquinone couple of the class exchanges two electrons and two protons. Anchoring groups appear in orange.

The five molecular classes were selected for plant-biosynthesized scaffolds with optical, redox, or coordination motifs relevant to semiconductor interfaces. Evidence from the screened PEC corpus supports chlorophylls, carotenoids, and polyphenols. Betalains and quinones are included as candidates supported by adjacent photochemical literature; their proposed PEC functions are distinguished from demonstrated results. Table 1 compares plant-derived molecules with synthetic sensitizers by interfacial role. Synthetic dyes permit broader tuning of excited-state and ground-state potentials and offer reproducible composition; a zinc porphyrin sensitizer achieved 12.3% efficiency in a DSSC [23]. Plant ligands offer complementary opportunities for multidentate coordination and solution deposition. Figure 3 presents representative structures, and Table 2 links their functional groups to anchoring, interfacial roles, and degradation. The following sections address intrinsic properties; PEC performance is examined in Section 4.

### 2.1. From Crude Extracts to Structurally Defined Molecules

Early studies on natural sensitizers relied predominantly on crude extracts from leaves, fruits, flowers, and peels [13,24,25,26]. Alongside the pigments such extracts carry alcohols and organic acids, and those accompanying species were proposed to act as co-adsorbents that suppress recombination [13]. A photocurrent measured under those conditions belongs to the ensemble and not to any one structure. Species, cultivar, harvest season, and extraction solvent each shift the composition, so results travel poorly between laboratories.

**Table 1:** Plant-derived molecules compared with the synthetic sensitizers they are proposed to replace, separated by the interfacial role under consideration.

Property	Ru Polypyridyl	Porphyrin/Organic D–π–A	Plant-Derived	What It Governs
**As a light harvester**				
Molar absorptivity	Moderate	High	High for chlorophylls and some pigment polyphenols; weak visible absorption for many phenolic ligands	Light harvesting
Independent tuning of excited- and ground-state potentials	Yes, by ligand design	Yes, by substituent design	Limited for native scaffolds; broader tuning through characterized derivatives	Energy-level matching
Anchoring	Carboxylate, monodentate	Carboxylate or cyanoacrylate	Catecholate and galloyl, multidentate	Contact stability
Batch-to-batch consistency	High	High	Source-dependent for extracts; assess purity and batch consistency for defined reagents	Reproducibility
Synthesis	Multistep; contains Ru	Multistep	Extraction or commercial reagent	Cost and criticality
Deposition	Organic solvent	Organic solvent	Ambient aqueous drop-casting	Process energy
**As a coordination scaffold and hole relay**				
Metal coordination network	Not a defining feature of the sensitizers compared here	Architecture-dependent; requires coordination-capable groups	Formed; crosslinks metal ions into a conformal layer	Catalytic overlayer
Conformal coverage of porous hosts	Limited	Limited	Demonstrated on nanoporous BiVO_4_	Interfacial contact
Benchmark photocurrent on BiVO_4_	Not applicable	Not applicable	3.08 to 6.2 mA cm^−2^ on BiVO_4_; superiority requires matched inorganic controls	Within-study benchmarking
Dominant degradation route	Ligand substitution, desorption	Photobleaching, aggregation	Demetalation, quinone oxidation, local-pH disassembly	Operating lifetime

**Table 2:** Intrinsic descriptors of plant-derived functional molecules relevant to photoelectrochemical interfaces. ROS, reactive oxygen species.

Class	Representative Molecules	Functional Motif	Anchoring or Coordinating Groups	Interfacial Role and Evidence Status	Dominant Degradation Pathway
Chlorophylls	Chlorophyll a, Cu-chlorophyllin, chlorin e_6_	Metallochlorin macrocycle	Carboxylate (after ester hydrolysis); central metal	Light harvesting; hole transport and protection in modified films	Demetalation under acidic conditions; photobleaching
Carotenoids	β-Carotene, lutein, crocetin	Conjugated polyene	Hydroxyl (xanthophylls); carboxylate (apocarotenoids)	Light harvester; triplet and ROS quencher	Radical oxidation; Z/E isomerization
Betalains	Betanin, betanidin, indicaxanthin	Betalamic acid chromophore	Carboxylate; catecholate (betanidin)	Candidate PEC sensitizer; evidence from dye-sensitized cells	Thermal and pH-induced degradation
Polyphenols	Caffeic acid, quercetin, tannic acid	Catechol and galloyl units	Catecholate chelation; carboxylate	Coordination scaffold; charge mediator; passivator	Oxidation to quinones
Quinones	Lawsone, juglone, alizarin	Quinone/hydroquinone couple	Hydroxy–carbonyl and catecholate chelation	Candidate PEC charge relay; no purified plant-quinone study in the screened corpus	Radical-mediated side reactions

Structural definition is a reporting attribute rather than an inclusion criterion. Crude extracts qualify when they function at a PEC electrode, and semi-synthetic derivatives qualify when they retain the native chromophore or ligand framework. Carbon dots prepared from chlorophyll [27] and the tannic-acid-derived carbon layer reported by Choi et al. [28] fall outside the molecular corpus because carbonization consumes the precursor; Choi et al. was retrieved by the search and appears among the screened exclusions in Table S4. They provide supporting evidence on precursor-derived materials. The TiO_2_–catechol complex [29] retains a plant metabolite at the interface and is included. In contrast, the Co-CAT framework on Cu_2_O@CuO [30] uses the synthetic ligand 2,3,6,7,10,11-hexahydroxytriphenylene (HHTP). Its exclusion follows from ligand identity, not from its photocathodic operation. Catechol and HHTP-based catecholate frameworks must therefore be distinguished.

Structural definition determines how specifically function can be assigned. A chlorophyll-based electrode prepared from a named compound differs from one prepared from an uncharacterized leaf extract. Source species, extraction conditions, and composition must accompany device metrics to permit reproducible comparisons. Inorganic films likewise require compositional and phase characterization; synthesis conditions alone do not establish the operating structure.

The five molecular readiness levels (MRLs) in Table 3 grade the reported definition of the molecular component. An undefined extract is assigned MRL 1, whereas a named single compound is assigned MRL 4. MRL 3 denotes a mixture with named major constituents, whereas MRL 2 denotes only class-level definition. Constituent identification and quantification by LC-MS or NMR are recommended for component-specific correlations but are not prerequisites for the identity-based MRL 3 assignment. The identity-based assignment does not independently verify purity or the molecular structure retained during operation. Table S3 records the assigned level and compound identity; it is not a study-by-study audit of analytical purity. MRL 5 requires characterization of an engineered derivative, beyond naming a modified material.

The scale specifies the scope of attribution supported by molecular identity. Crude extracts support ensemble-level observations, whereas defined compounds permit testing of molecule-specific hypotheses. Mechanistic assignment additionally requires suitable controls, charge-transfer measurements, and evidence of the operating chemical structure. Neither compound identity nor purity alone establishes a mechanism.

Of the 26 retained studies, 19 were assigned MRL 4 from the reported compound identity, one was assigned MRL 3, and six were assigned MRL 1 for undefined extracts (Figure 1f; Table S3). No study was assigned MRL 2 or 5 under the applied rules. The absence of MRL 5 assignments does not establish that no modified derivative was used. The distribution instead indicates that mixture profiling and characterization of engineered components require more explicit reporting.

Complex natural sources need not be abandoned to reach structural definition. Multimodal spectroscopy combined with quantum chemical calculation can resolve individual constituents within an extract, with principal component analysis, t-distributed stochastic neighbor embedding (t-SNE), or uniform manifold approximation and projection (UMAP) used to group congeners during the assignment. What emerges is a library of identified candidates, each of which can then be tested for a specific interfacial function (Section 3.4).

### 2.2. Chlorophylls and Semi-Synthetic Tetrapyrrole Derivatives

Chlorophylls consist of a Mg-coordinated chlorin macrocycle bearing a long phytyl ester chain [31]. The extended π system produces an intense Soret band near 430 nm and a Q_y_ band, the lowest-energy π→π* transition of the macrocycle, near 660 nm, which together cover the blue and red regions of the solar spectrum. The molar absorption coefficient of the Q_y_ band of chlorophyll a exceeds 7 × 10^4^ M^−1^ cm^−1^. The isocyclic ring and the ester groups provide oxygen-containing sites for surface interaction. Hydrolysis of the phytyl ester exposes a carboxylic acid that enables covalent anchoring to oxide surfaces.

Acid alone displaces the central Mg ion of chlorophyll, leaving pheophytin with shifted absorption and altered redox properties. Cu or Zn in place of Mg gives a far more robust metallochlorin. Saponification takes a different route and yields water-soluble chlorophyllins carrying several carboxylates, while chlorin e_6_ and pheophorbide a keep the native macrocycle intact and come with carboxylic acid handles already attached.

How far the surroundings can shift that chemistry is clear from PSII itself. Its primary donor P_680_ reaches an oxidation potential near +1.2 V vs. the normal hydrogen electrode (NHE), enough to drive water oxidation through the tyrosine relay, whereas monomeric chlorophyll in solution oxidizes far below that value. Anchoring chemistry, central-metal substitution, and the local dielectric environment offer analogous levers at a semiconductor surface, and PEC designs have scarcely exploited them.

Chlorophyll-based components have been integrated into BiVO_4_ photoanodes, Si photocathodes, and polaronic oxides, and Section 4.1, Section 4.2, Section 4.3 and Section 4.4 analyze the corresponding interfacial mechanisms.

### 2.3. Carotenoids

Carotenoids are C_40_ isoprenoids built on a conjugated polyene chain of typically 9 to 11 double bonds [32]. Their strongly allowed S_0_→S_2_ transition absorbs between approximately 400 and 500 nm, filling the window that chlorophyll leaves open. Relaxation is the difficulty. The S_2_ state of β-carotene reaches an optically dark S_1_ state within a few hundred femtoseconds, and S_1_ itself decays within about 10 ps, so electron injection has to be faster still and demands intimate coupling to the semiconductor. In photosynthesis the same molecules quench chlorophyll triplets and singlet oxygen, a photoprotective role that bears directly on device stability.

Anchoring capability varies across the class. β-Carotene is a pure hydrocarbon and interacts with surfaces mainly through van der Waals and π interactions. Xanthophylls such as lutein and zeaxanthin carry terminal hydroxyl groups, whereas apocarotenoids such as bixin and crocetin present carboxylic acids suitable for covalent binding.

Beyond light harvesting, the conjugated polyene conducts charge along its axis, and carotenoids have served as molecular wires and secondary electron donors in artificial photosynthetic assemblies. A hole-relay function may therefore suit carotenoids at PEC interfaces better than direct sensitization, particularly when combined with their capacity to quench triplet states and ROS.

The polyene chain also constitutes the principal site of degradation. Initial oxidation rates measured with peroxyl and hydroxyl radicals decreased in the order lycopene > β-carotene ≈ zeaxanthin > echinenone > astaxanthin ≈ canthaxanthin, and substitution at the allylic C-4 and C-4′ positions slowed the reaction, which implicates hydrogen abstraction alongside radical addition [33]. Terminal functionalization thus provides a structural lever for oxidative stability. Excited-state sensitization alters the polyene as well: riboflavin drove Z-isomerization of (all-E)-carotenoids by triplet–triplet energy transfer and raised the total Z-isomer fraction of lycopene by more than 50% [34], and triplet sensitization by tetraphenylporphyrin, enabled by spin–vibronic intersystem crossing, raised the quantum yield of prolycopene isomerization from 0.003 to 0.58 [35]. Direct PEC evidence is limited to a single screened study, in which a β-carotene coating modified the photoelectrochemical response of hematite nanoarchitectures [36]. β-Carotene-sensitized anatase [37] was measured photoelectrochemically, although for sunlight response rather than water splitting, so it lies outside the screened corpus. Evidence from chlorin-modified Pt/TiO_2_ [38], carotenoid–MXene composites [39], and a semi-synthetic chlorophyll–carotenoid dyad [40] was obtained under photocatalytic conditions and serves here as transferable molecular evidence.

### 2.4. Betalains

Betalains are tyrosine-derived pigments that share betalamic acid as the common chromophore [41]. Condensation of betalamic acid with cyclo-DOPA yields red-violet betacyanins such as betanin, whereas condensation with amino acids or amines yields yellow betaxanthins such as indicaxanthin. Betanin absorbs maximally near 535 nm. Betalains replace anthocyanins in most families of the Caryophyllales, and anthocyanins have not been detected in betalain-producing species [41].

Betanin combines a molar absorption coefficient of 65,000 M^−1^ cm^−1^ at 535 nm with three carboxylic acid groups, a combination attractive for anchoring to TiO_2_ and ZnO [42]. The betanidin aglycone additionally exposes a free catechol, so removal of the C5 glucose converts a carboxylate-only anchor into a chelating one. A betanin-sensitized solar cell delivered 2.42 mA cm^−2^ and 0.44 V, with peak incident photon-to-current efficiency (IPCE) values of 8% for betanin and 14% for the yellow betaxanthin fraction [42].

The screened Scopus corpus contained no PEC study on purified betalains (Figure 1d). Betanin is most stable in mildly acidic solution, around pH 4–5, and degrades under heating, illumination, and oxygen exposure. Acylation and glycosylation patterns alter the stability of natural betacyanins and offer structural handles for selection, while abundant sources such as red beet and dragon fruit peel favor low-cost supply. The class represents an underexplored opportunity for sensitization of wide-bandgap photoanodes.

### 2.5. Polyphenols: Phenolic Acids, Flavonoids, and Tannins

Polyphenols form the most diverse class and include phenolic acids such as caffeic and gallic acid, flavonoids such as quercetin and the anthocyanins, and hydrolyzable tannins such as tannic acid. The catechol and galloyl units shared by the class chelate transition-metal ions with high affinity. Coordination of tannic acid with Fe^3+^ yields metal–phenolic networks (MPNs) that assemble into conformal films on diverse substrates in a single step [43].

Quercetin illustrates the structural features that favor coordination. Its 3-hydroxy–4-keto, 5-hydroxy–4-keto, and 3′,4′-catechol motifs provide three distinct chelation sites whose occupancy depends on pH and metal ion, and the 3-hydroxy group facilitates metal binding and stabilizes active sites [20]. Anthocyanins, by contrast, are valued mainly as chromophores. Their flavylium color depends strongly on pH, and their stability is sensitive to pH, temperature, and oxygen [13].

Polyphenols are intrinsically redox active. Catechol units undergo two-electron, two-proton oxidation to o-quinones with an approximate standard reduction potential of +0.79 V vs. NHE at pH 0 [44], a property that enables hole scavenging and radical quenching. The same property introduces a risk, because oxidation of the ligand competes with water oxidation. Section 5.3 addresses how to discriminate between the two processes.

Deprotonation of phenolic groups, with first p*K*_a_ values typically between 7 and 9, controls metal-binding strength, surface charge, and oxidation potential at once. Electrolyte pH thus acts as a single design variable that simultaneously sets the coordination, charge, and redox behavior of polyphenol layers, and it should be optimized jointly with the host semiconductor.

### 2.6. Quinones

Plant quinones comprise benzoquinones such as plastoquinone, naphthoquinones such as lawsone from henna and juglone from walnut, and anthraquinones such as alizarin and emodin [45]. Their quinone/hydroquinone couple undergoes reversible two-electron, two-proton reactions, and ring annulation and substituents tune the redox potential over a wide range, from approximately +0.70 V vs. NHE at pH 0 for p-benzoquinone [44] to progressively lower values for naphthoquinones and anthraquinones. Potentials quoted against the standard hydrogen electrode in the source literature lie on the same scale at unit activity. In PSII, plastoquinones serve as electron acceptors that stabilize charge separation.

Hydroxyquinones combine a redox core with a chelating motif. Lawsone and juglone present a salicylate-type hydroxy–carbonyl pair, whereas alizarin offers a 1,2-dihydroxy catecholate adjacent to the C9 carbonyl. Lawsone served as a co-sensitizer with betanin in plasmon-enhanced solar cells [46], and a 1,4-naphthoquinone-based dye was examined experimentally and theoretically on ZnO photoanodes [47]. The screened corpus likewise contained no PEC water splitting study on a purified plant quinone (Figure 1d). Their reversible redox chemistry nevertheless positions quinones as candidate charge relays between semiconductors and catalysts, as in natural photosynthesis.

## 3. Molecular Design Principles for Photoelectrochemical Interfaces

What a molecule does at a photoelectrode is set by its energetic alignment with the host, by the chemistry of its surface binding, and by how long it survives under illumination and bias. Section 3.4 turns to computation and data-driven tools, which can accelerate selection among the many natural candidates.

### 3.1. Energy-Level Alignment and Excited-State Dynamics

For sensitization of an n-type photoanode, the excited-state oxidation potential of the molecule must be more negative than the conduction-band potential of the semiconductor. The ground-state oxidation potential must, in turn, be sufficiently positive to accept electrons from water or from a water oxidation catalyst. Injection also has to outcompete excited-state decay. Chlorophylls and chlorins, with nanosecond singlet lifetimes and accessible triplet states, satisfy the kinetic condition more readily than carotenoids (Section 2.3).

Hole-transfer mediation imposes different requirements. The highest occupied level of the molecule or metal–molecule complex should lie between the valence band edge of the absorber and the water oxidation potential, creating a thermodynamic cascade for holes. The mediator must also accept holes faster than surface recombination consumes them. The tannic acid–Co layer on BiVO_4_ fulfilled both conditions, since photogenerated holes transferred directly to the layer and oxidized Co^2+^ to catalytically active Co^3+^ [7].

Net quinone/phenol reactions and individual one-electron steps describe different thermodynamic quantities. Proton-coupled two-electron potentials must be specified with the protonation regime, solvent, and reference electrode; they do not establish the potential or kinetics of a one-electron hole relay. The first anodic wave of betanin was reported near +0.62 V vs. NHE at pH 7.4, corresponding to approximately +1.06 V vs. RHE [42]. An anodic peak is not a reversible formal potential. Productive Co^2+^/Co^3+^ mediation was reported for a tannic acid-Co layer [7], supporting a metal-centered pathway for that system without establishing charge localization in every polyphenol hybrid.

**Figure 4:**
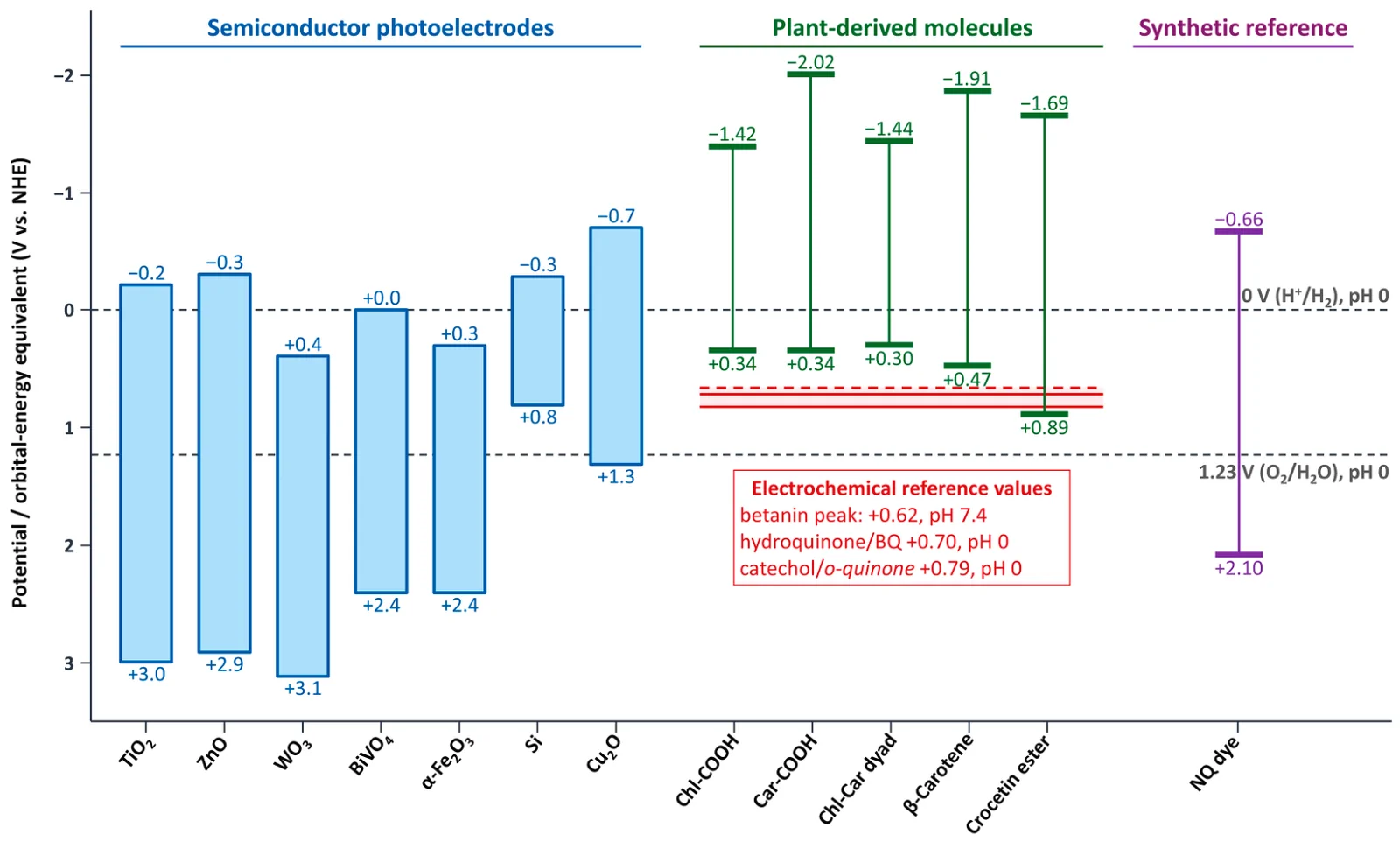
Energy alignment of plant-derived molecules with representative photoelectrode hosts on the NHE scale. Blue bars show representative semiconductor band positions, and gray dashed lines mark the hydrogen and oxygen evolution potentials at pH 0. Green and purple levels show calculated frontier orbital energies of plant-derived molecules and the synthetic naphthoquinone reference, respectively [39,40,47]. Orbital energies were converted using E = −E(vacuum) − 4.44 and represent NHE-equivalent estimates. Solid red lines indicate approximate two-electron reference potentials of the hydroquinone/benzoquinone and catechol/o-quinone couples at pH 0 [44]. The dashed red line marks the betanin anodic peak at +0.62 V vs. NHE at pH 7.4 [42]. The peak is distinct from an equilibrium redox potential. Band positions depend on material and measurement conditions; alignment under operating conditions requires a common reference scale and pH. Table S5 lists the molecular values and their sources.

Figure 4 separates calculated orbital-energy estimates from measured electrochemical quantities. The sensitizer excited-state oxidation potential must be more negative than the conduction-band potential of the selected host for favorable electron injection. A hole relay requires a relevant oxidation potential between the water oxidation potential and the host valence-band potential, with additional kinetic and overpotential requirements. No single potential interval applies to every host. One-electron phenoxyl or semiquinone steps must be measured independently of net two-electron couples, since they can lie well above the net two-electron value and a potential above the water oxidation potential does not by itself exclude hole relay; feasibility also depends on alignment with the host valence band and the kinetics of charge transfer and competing reactions. No one-electron potential was reported for the systems compiled in this review. Potentials of proton-coupled couples are independent of pH on the RHE scale only while the proton-coupled pathway holds, which for phenolic ligands fails above the first pK_a_ of 7 to 9 (Section 2.5). Metal coordination changes the relative stabilization of the oxidized and reduced ligand states and can alter ligand deprotonation and metal-ligand charge distribution. The resulting redox response depends on the metal, coordination environment, and pH; coordination does not necessarily shift the potential in a direction favorable for water oxidation. Metal- and pH-dependent shifts in the ligand anodic peak have been measured for catechol-terminated monolayers [48], although such peak shifts are not equivalent to changes in a reversible formal potential. Potentials measured for free ligands therefore cannot be assigned directly to the coordinated layer. Orbital energies cannot substitute for those measurements.

Coordination can also reshape the electronic structure of the host. Density functional theory (DFT) calculations on quercetin–NiCo-functionalized BiVO_4_ revealed an upshift of the O p-band center from −2.708 to −2.369 eV, indicating stronger O 2p–metal d hybridization at the interface [20]. Molecular overlayers act as electronic modifiers of the semiconductor surface, not merely as passive light absorbers.

### 3.2. Anchoring Groups and Interfacial Binding Modes

Most plant molecules reach an oxide surface through a carboxylate, which binds in monodentate, bidentate chelating, or bridging modes. The hold weakens in neutral and alkaline electrolytes, and the molecule desorbs over extended operation. A catechol does better. It forms a bidentate complex with surface metal cations and can generate an interfacial charge-transfer state that extends absorption beyond the molecular spectrum. Hydroxy–carbonyl motifs, found in lawsone and juglone, chelate through the same oxygen pair.

Surface charge governs anchoring under operating conditions. The point of zero charge of TiO_2_ lies near pH 6, so electrostatic interactions with anionic anchors change across that pH region. Desorption additionally depends on binding mode, surface composition, and competing electrolyte species. Phosphonate and hydroxamate anchors generally resist hydrolysis in water better than carboxylates, and for that reason they dominate synthetic dye-sensitized photoelectrodes. Betalain sensitization illustrates the consequence: betanin adsorbed only weakly on untreated TiO_2_ and required acid pretreatment of the film to reach appreciable loading [42]. For plant molecules, multidentate coordination networks offer an alternative route to aqueous stability that requires no synthetic modification.

Coordination-mediated binding provides a fourth mode. Metal ions such as Fe^3+^, Co^2+^, or Ni^2+^ crosslink polyphenol ligands into continuous films [43]. In quercetin–NiCo-functionalized BiVO_4_, spectroscopic analysis identified metal–phenolic C–O–M linkages together with Ni/Co–O–V interfacial bonds [20]. Multidentate, crosslinked binding of this type is expected to resist desorption more effectively than single-point anchoring.

Non-covalent interactions complete the repertoire. Hydrophobic carotenoids lack strong anchors and depend on π interactions or electrostatic contact. Terminal groups govern such contact even without covalent anchoring. Among three carotenoids deposited on Ti_3_C_2_T*_x_* MXene, the planar crocetin diethyl ester achieved the closest interfacial contact and 84.4 μmol h^−1^ g^−1^ of hydrogen, compared with 45.9 μmol h^−1^ g^−1^ for hydrocarbon β-carotene [39]. Semi-synthetic introduction of phosphonate or hydroxamate groups opens a route to stronger anchoring, although such modifications have not been explored for plant scaffolds in PEC systems.

### 3.3. Degradation Pathways and Stabilization Strategies

Instability represents the most serious objection to plant-derived molecules. Natural dye sensitizers dissolve or degrade in aqueous electrolytes, and protective conductive polymer layers have been proposed to retain them [13]. Four degradation routes dominate: demetalation of chlorophylls under acidic conditions, radical oxidation and isomerization of carotenoids [33,34], pH- and oxygen-induced degradation of anthocyanins and betalains [13], and oxidation of polyphenols to quinones under anodic bias.

**Figure 5:**
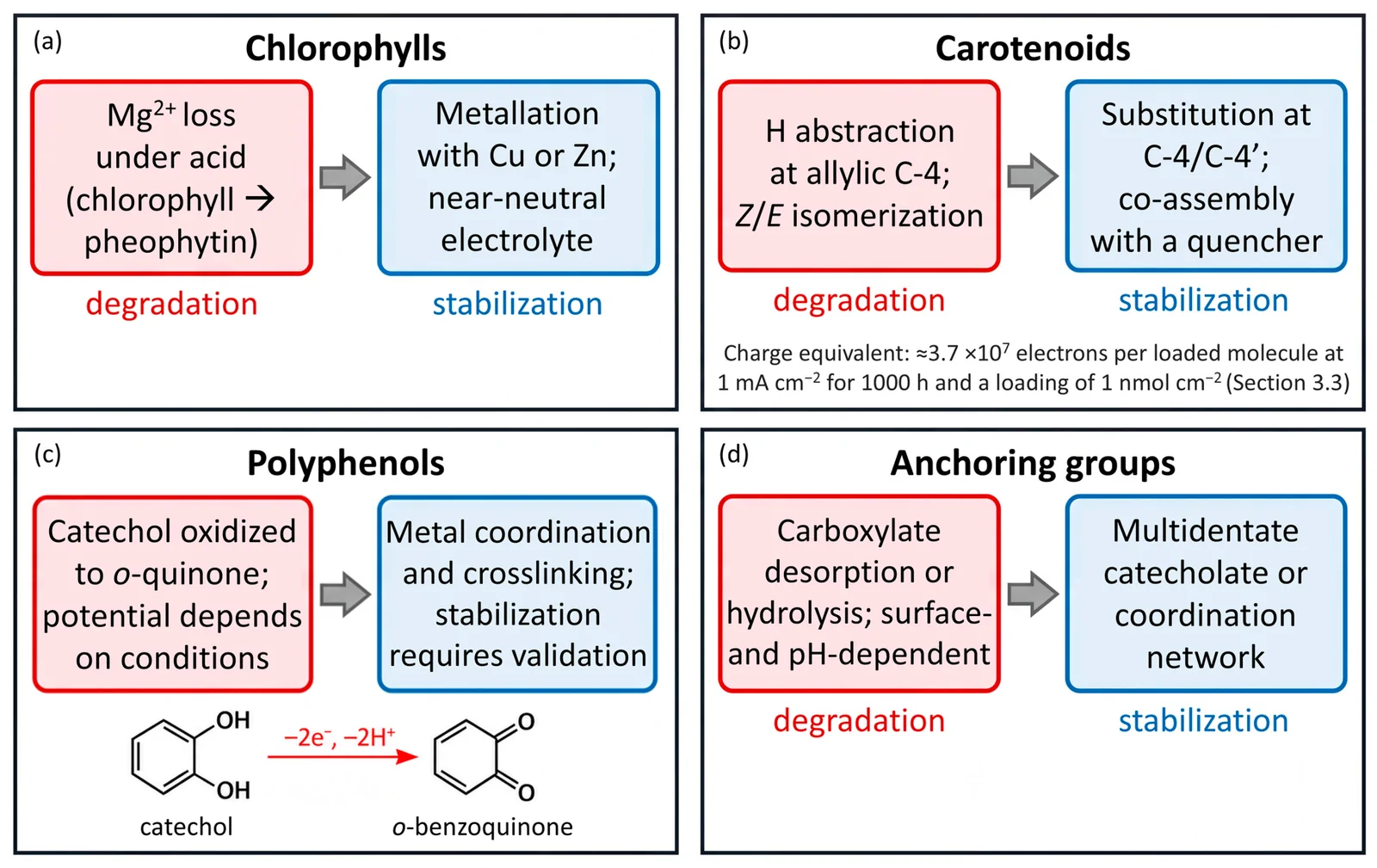
Degradation risks and candidate stabilization strategies. (**a**) Acid-induced chlorophyll demetalation and potential mitigation by metal substitution or electrolyte control. (**b**) Carotenoid oxidation and isomerization, with substitution or protective co-assembly as possible remedies. (**c**) Phenolic oxidation and coordination-based stabilization; metal-centered hole transfer requires system-specific evidence. (**d**) pH-dependent anchor hydrolysis or desorption and possible stabilization through multidentate binding. The point of zero charge describes surface charging, not a universal desorption threshold. At 1 mA cm^−2^ and 1 nmol cm^−2^, 1000 h corresponds to approximately 3.7 × 10^7^ electrons passed per molecule in the initial inventory. That normalization is a survival requirement for spectator ligands.

Stabilization strategies act at molecular and interfacial levels, and Figure 5 pairs representative degradation risks with candidate remedies: metallation against demetalation, terminal substitution against allylic abstraction, metal crosslinking against quinone formation, and multidentate coordination against desorption. Section 2.3 and Section 3.3 document the behavior that motivates the pairing: the structure dependence of carotenoid bleaching, and the partial loss of activity across recycling runs. Metallation with Cu or Zn protects the chlorin core. Co-assembly with carotenoids introduces intrinsic photoprotection, as exploited in a covalently linked chlorophyll–carotenoid dyad [40]. Crosslinking through metal coordination converts soluble ligands into insoluble networks [43].

Molecular layers can also stabilize the semiconductor itself: chlorophyll films suppressed vanadium loss from BiVO_4_ (Section 4.2) [49]. Electrolyte engineering provides a complementary lever, as illustrated by tuning of the electrolyte composition to extend the photostability of BiVO_4_ [50]. Combining protective molecular layers with tailored electrolytes remains largely untested for plant-derived systems.

A simple charge balance quantifies the required molecular robustness. The inventory-normalized electron count is calculated as N_e_ = q/(FΓ), where q is the cumulative charge per unit area, F is the Faraday constant, and Γ is the initial molecular loading in mol cm^−2^. This quantity does not establish that every electron passes through the ligand. Operation at 1 mA cm^−2^ for 1000 h passes 3.6 × 10^3^ C cm^−2^, equivalent to approximately 3.7 × 10^−2^ mol of electrons per square centimeter. The same balance fixes what 100 h of operation at that current and coverage demands, namely 3.7 × 10^6^ electrons per initially loaded molecule, which clears the 10^6^ floor adopted later as the near-term reporting target. At a molecular loading of 1 nmol cm^−2^, the total charge corresponds to approximately 3.7 × 10^7^ electrons per molecule in the initial inventory. Under a simple independent-event model, a degradation probability of 10^−6^ per event would leave a negligible surviving fraction. For spectator ligands, normalized charge describes a survival demand rather than actual molecular turnover. The calculation illustrates the stringent durability demand under the stated model; it does not predict the lifetime of a spectator ligand or establish the mechanism of experimental degradation.

Molecular oxidation is only one source of deactivation. Anchor hydrolysis and desorption can reduce coverage, while metal leaching can disrupt coordination networks. Anodic reconstruction may generate oxyhydroxide-like active sites, as suggested for the quercetin–NiCo layer [20]. Retained organic signals after operation do not establish that the original network remained the active phase. Bubble-driven delamination is another possible failure mode and requires direct examination. Loading measurements, electrolyte analysis, and operando spectroscopy are needed to distinguish these processes. In supporting photocatalytic studies, carotenoid-containing systems also lost activity during cycling or prolonged illumination [38,39]; those observations indicate potential degradation pathways without defining PEC lifetimes.

### 3.4. Computation- and Data-Driven Molecular Identification and Design

DFT and its time-dependent extension provide frontier orbital energies, excitation energies, adsorption geometries, and site-resolved reactivity descriptors, and they do so before any molecule is extracted or any electrode is built. The approach matters most for plant-derived molecules because natural product space is far larger than any experimental screening campaign can cover. Combined experimental and time-dependent calculations placed the frontier levels of a 1,4-naphthoquinone dye at −6.54 and −3.78 eV, which yields a 0.41 eV driving force for injection into ZnO and 1.55 eV for regeneration, and the resulting cell reached 1.07% efficiency [47]. Quantum chemical analysis similarly identified triplet–triplet energy transfer as the route of carotenoid photoisomerization [34]. Periodic slab calculations further resolved site-specific catalytic activity in MPN overlayers on BiVO_4_, where projected densities of states, d-band and O p-band centers, charge-density differences, and adsorption free energies together identified Ni as the active site and Co as an electronic promoter [20]. Frontier orbital analysis of a chlorophyll–carotenoid dyad likewise located the highest occupied orbital on the polyene and the lowest unoccupied orbital on the macrocycle, which predicted the direction of intramolecular charge transfer later confirmed by wavelength-resolved hydrogen evolution [40].

Methodological choices strongly affect predictive value. Generalized gradient functionals underestimate band gaps and misplace molecular levels relative to oxide band edges, so hybrid functionals or DFT+U corrections are required for reliable alignment. Range-separated functionals describe the charge-transfer excitations of donor–acceptor pigments more reliably in time-dependent calculations, and published benchmarks give typical error bars of 0.2 to 0.3 eV for organic chromophores, which is comparable to the injection driving forces under discussion [51]. Reporting the functional, basis set, solvation model, and the reference against which a level was calibrated is therefore as important as reporting the predicted value itself. Solvation, pH-dependent protonation of phenolic groups, and explicit interfacial water can each shift predicted levels by several hundred millivolts, and their omission can reverse the predicted direction of charge transfer.

The same structure–property discipline governs other fields that engineer small organic molecules for a photophysical function, where substituent position, conjugation length and aggregation state set the emission wavelength, quantum yield and photostability of a probe [52]. Transferring that discipline to photoelectrochemical interfaces requires the same prerequisite. A molecule must be identified before its function is assigned. The natural product chemical space far exceeds what calculation alone can address. Open databases such as COCONUT collect hundreds of thousands of natural product structures [53]. Descriptor-based screening can filter such databases for chelating motifs, anchoring groups, and frontier orbital windows before any quantum chemical calculation. Screening campaigns of comparable ambition are established elsewhere in energy research: the Harvard Clean Energy Project characterized millions of candidate structures for organic photovoltaics by automated quantum chemistry and released the results as a public database [54]. An equivalent effort has not been attempted for natural products in PEC water splitting, even though the search space is bounded by biosynthesis and therefore smaller and better annotated than combinatorial chemical space. Descriptors already exist for each of the four interfacial roles of Figure 2. Light harvesting calls for conjugation length and oscillator strength, charge relay for oxidation potential and reorganization energy, coordination scaffolding for denticity and metal-binding constants. Passivation requires descriptors of surface binding, trap-state modification, and resistance to corrosion. Radical-scavenging rates describe photoprotection and do not alone establish electronic passivation.

Autonomous experimentation can circumvent the shortage of training data. A mobile robot working from a batched Bayesian search improved photocatalytic hydrogen production sixfold across 688 experiments in eight days, selecting beneficial components and rejecting others without human intervention [55]. A genetic algorithm coupled to a neural network has selected combinations of natural leaf extracts for water splitting electrodes [56]. Compositional uncertainty did not prevent the selection from converging. Plant-derived interfaces suit such loops because deposition is usually a dip-coating or drop-casting step at room temperature, and the measured objective can be the verified quantity of Section 5 rather than the raw photocurrent.

Data-driven tools also serve identification. Spectral fingerprints from infrared, nuclear magnetic resonance, and photoluminescence spectroscopy, combined with computed spectra and dimensionality reduction, can assign unknown constituents of plant extracts. Integration of identification and design closes the loop between crude natural sources and structurally defined interfacial components. A practical pipeline runs from database filtering to quantum chemical ranking, then to automated synthesis of the shortlisted anchoring derivatives, and finally to an autonomous PEC measurement loop that reports Faradaic efficiency (FE) and molecular turnover. Each stage produces labeled data for the next, and the reporting items proposed in Section 5 define the labels. The scarcity of such data, rather than the absence of suitable molecules, currently limits the field.

## 4. Interfacial Hybridization: Functional Roles and Architectures

Hybrid photoelectrodes are classified below by the function that the plant-derived component performs at the interface. Figure 2 presents the classification: five molecular classes on the left, the four interfacial roles in the center, and the counterpart of each role in PSII on the right. The mapping is many-to-many, since polyphenols serve three roles and carotenoids two, so the molecular class alone does not determine the function. Table 4 translates the scheme into host-specific design rules by listing, for five classes of semiconductor, the property that limits performance, the molecular remedy that addresses it, and the structural requirement that the molecule must satisfy. A benchmarking table in Section 5.4 then compiles representative performance data, dominated by BiVO_4_ photoanodes, the host for which enough studies exist to support comparison.

Among the 26 retained studies, 11 were assigned light harvesting as the primary role, eight charge-transfer mediation, and seven coordination-scaffold formation (Figure 1e). The copper-substituted chlorophyll film of Lv et al. was classified as a charge-transfer mediator, consistent with its hole-transport role [49]. No retained study targeted passivation alone. Coordination-scaffold studies appeared from 2018 onward, whereas the catechol sensitization study appeared in 2014 [29]. The distribution shows growing attention to charge transfer and catalytic interfaces.

### 4.1. Molecular Photosensitization

Sensitization extends the spectral response of a host through electron injection from photoexcited molecules, and matters most for wide-bandgap oxides. TiO_2_ has served as the dominant host, and chlorophyll derivatives, anthocyanins, carotenoids, and betalain extracts emerged as the most effective natural sensitizers, with monochromatic photon-to-current conversion exceeding 60% in selected cases [13]. Sensitization by plant molecules also occurs outside the laboratory: natural surface coatings enriched in anatase and β-carotene motivated experiments in which β-carotene raised the photocurrent of anatase by 64–66% and added absorption between 425 and 550 nm [37]. Chlorophyll a on ZnO nanorods improved the water splitting response of the oxide [57], and dyes extracted from basil, spinach, and pomegranate sensitized sputtered ZnO films [24]. Six of the eleven studies assigned to light harvesting used compositionally undefined extracts.

Sensitization of visible-light absorbers follows a different logic. BiVO_4_ already absorbs below approximately 520 nm, so a sensitizer contributes mainly through absorption at longer wavelengths, such as the chlorophyll Q_y_ band. Chlorophyll-sensitized BiVO_4_ was applied to both solar water splitting and CO_2_ conversion [58], and natural sensitizers also enhanced water splitting on polaronic nanomaterials [59]. A chlorophyll/Cu_2_O heterostructure similarly raised the applied bias photon-to-current efficiency (ABPE) of a water splitting electrode, with the chromophore applied as an unfractionated algal extract [60].

Chlorophyll a sensitization lowered the photocurrent of BiVO_4_ by 14%, whereas copper chlorophyllin increased it by a factor of 1.82 [58]. The hydrogen evolution rate increased from 2.06 to 5.43 μmol h^−1^ cm^−2^, with FE above 86% for both gases. Contact angles decreased from 69.3° to 55.9° with the derivative and increased to 98.9° with chlorophyll. Surface wettability therefore contributed to the contrasting responses. The observations do not exclude simultaneous optical or electronic effects; wavelength-resolved measurements are needed to separate those contributions.

The optical contribution depends on molecular coverage. For a chlorophyll layer with ε = 7.6 × 10^4^ M^−1^ cm^−1^, the absorbed fraction is 1 − 10^−1000εΓ^, where Γ is the loading in mol cm^−2^. Peak absorption is approximately 1.7%, 8.4%, and 16.1% at 0.1, 0.5, and 1 nmol cm^−2^, respectively. A molecular footprint of 1–2 nm^2^ corresponds to approximately 0.08–0.17 nmol cm^−2^, so 1 nmol cm^−2^ exceeds a compact monolayer. The 6–12 nm coordination overlayers reported for tannic acid–Co networks (Section 4.2) correspond to approximately 0.5–1 nmol cm^−2^ of tannic acid units at a film density near 1.4 g cm^−3^, so the reference loading describes a layer several molecules thick. Integrating the ASTM G173 global-tilt photon flux from 640 to 680 nm gives 2.98 mA cm^−2^ at unit photon-to-electron conversion [61]. Applying 1.7% absorption throughout that interval gives an upper bound of approximately 0.05 mA cm^−2^ at 0.1 nmol cm^−2^. The estimate overstates absorption away from the peak and assumes complete charge collection. It applies only to the specified Q_y_ interval and coverage. Contributions from the Soret band and charge-transfer absorption must be included before interpreting broadband photocurrent enhancement. Porous electrodes and thick overlayers require measured loading and wavelength-dependent absorption rather than a compact-monolayer assumption. The calculation is documented in the Supplementary Materials.

**Figure 6:**
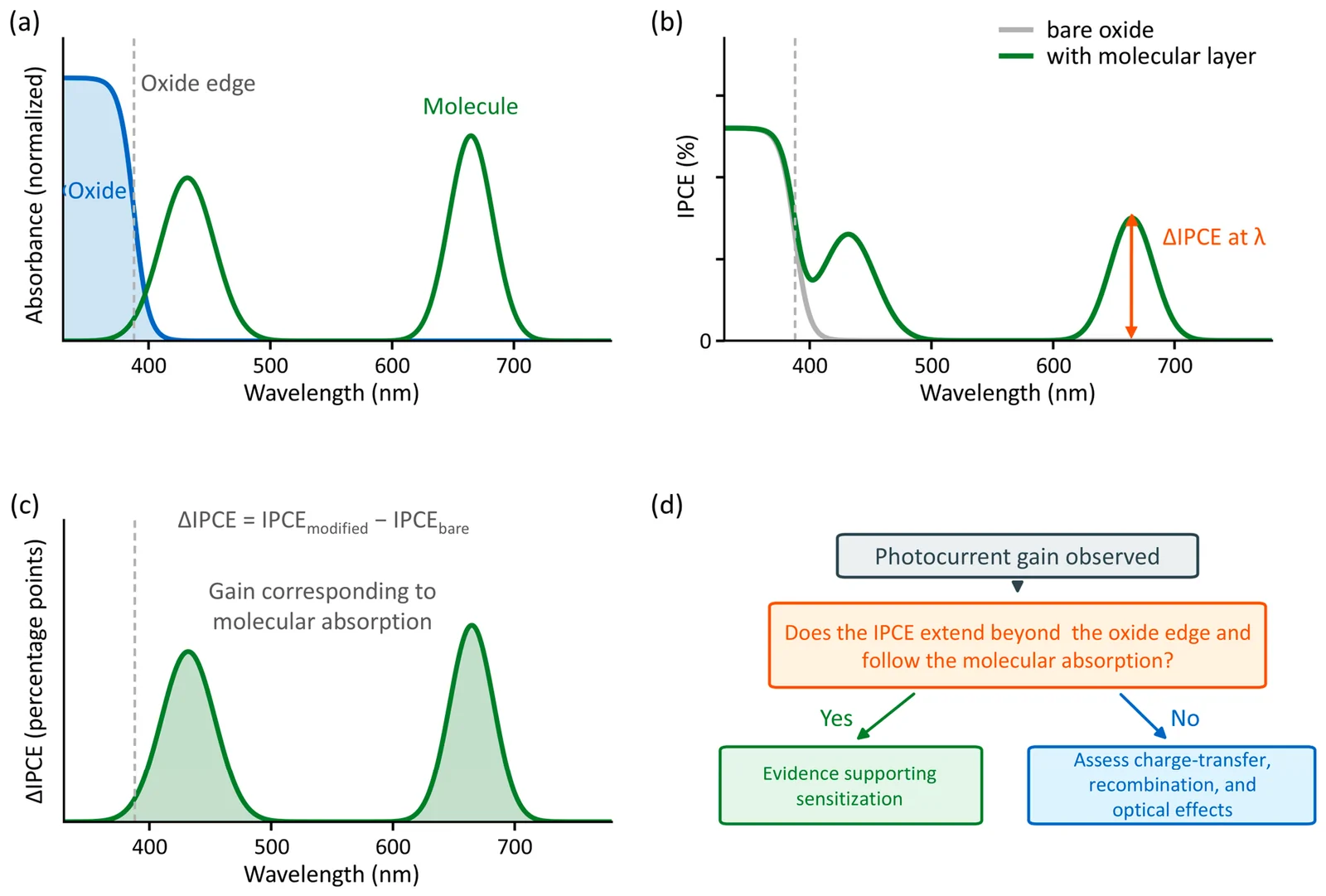
Spectral diagnostics of molecular sensitization. (**a**) Schematic absorption profiles of a wide-bandgap oxide and a molecular layer. (**b**) Schematic IPCE spectra before and after modification. (**c**) Spectral photocurrent enhancement expressed as ΔIPCE. (**d**) Evidence required for mechanistic attribution. Curves are illustrative and are not experimental data. The 8% and 14% IPCE maxima in Ref. [42] were obtained in betanin- and betaxanthin-sensitized DSSCs with an iodide/triiodide electrolyte. Ref. [37] provides supporting evidence for enhanced optical absorption and photocurrent in carotene-sensitized anatase. Neither example alone establishes sustained PEC water oxidation by the molecular layer.

Figure 6 illustrates wavelength-resolved tests of sensitization. An IPCE increase beyond the absorption edge of the host that follows molecular absorption supports a light-harvesting contribution. Overlapping absorption can obscure that signature. The spectral difference, ΔIPCE, should therefore be interpreted with measured absorption, loading, and charge-transfer data. ABPE is evaluated separately as a function of applied bias under a specified illumination spectrum.

Sensitized systems run into the same limitations again and again. An oxidized sensitizer has to be regenerated faster than it degrades, and that means coupling it to a water oxidation catalyst. Where the sensitizer came from a crude extract, the identity of the active chromophore is unknown to begin with. Worse, photocurrent enhancements are frequently reported with no spectral evidence at all, such as an IPCE spectrum matching the molecular absorption, and a sensitization claim means little until that evidence is supplied.

### 4.2. Hole-Transfer Mediation and Molecular Bridging to Cocatalysts

**Figure 7:**
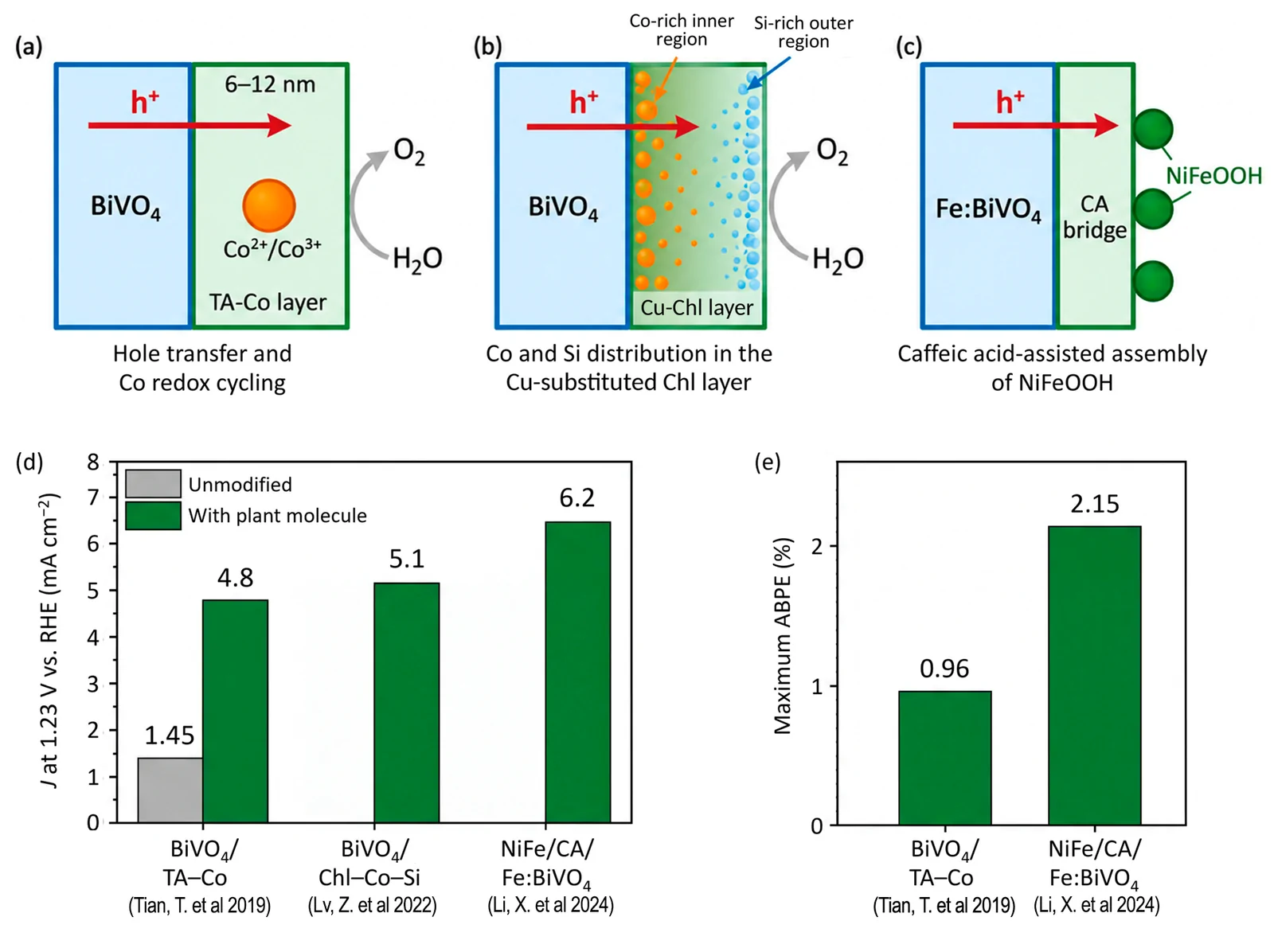
Representative molecularly modified BiVO_4_ photoanodes. (**a**) Tannic acid-Co layer [7]. (**b**) Copper-substituted chlorophyll film (Cu-Chl) with Co and Si distributed across the interface [49]. (**c**) Caffeic acid bridge between Fe-doped BiVO_4_ and NiFeOOH [19]. (**d**) Reported photocurrents at 1.23 V vs. RHE. The bare-host value is shown only for Ref. [7]; missing comparator bars do not represent zero current. (**e**) Maximum ABPE of the two systems that report it, obtained at source-specific biases, 0.74 V for Ref. [7] and 0.65 V for Ref. [19]; neither source reports a bare-host ABPE. Different hosts, morphologies, and operating conditions preclude attribution of cross-study differences solely to the plant ligand.

Molecular layers on visible-light absorbers most often function as hole-transfer mediators and not as sensitizers. Figure 7a summarizes a representative case, in which a tannic acid–Co network (TACo) was deposited on nanoporous BiVO_4_ by one-step coordination assembly without special equipment [7]. Photogenerated holes transferred directly from BiVO_4_ to the TACo layer and oxidized Co^2+^ to Co^3+^ sites capable of oxidizing water to O_2_ [7]. The photocurrent reached 4.8 mA cm^−2^ at 1.23 V vs. RHE, approximately three times that of bare BiVO_4_. ABPE reached 0.96% at 0.74 V vs. RHE [7]. Microscopy, spectral response and photoelectron spectra reported in the same study support both contributions: the layer is 6–12 nm thick and amorphous, the IPCE rises from below 20% to 47.3%, and Co^3+^ appears after 1 h of illumination [7]. The study therefore separated a thermodynamic contribution from hole extraction and a kinetic contribution from catalytic Co sites. A related tannic acid–Co layer on oxygen-vacancy-rich BiVO_4_ displayed the same dual function [16].

The same chemistry transfers to hematite. Gallic acid bridging a tannin–Co complex on Ti-doped Fe_2_O_3_ raised the photocurrent from 0.33 to 3.3 mA cm^−2^ at 1.23 V vs. RHE in 1 M KOH, shifted the onset by 210 mV and raised the injection efficiency from 7.6 to 75%, which extends the metal–phenolic strategy beyond BiVO_4_ to a second visible-light absorber. Gas chromatography gave an FE above 93% for oxygen with the evolved gases in the expected 1:2 ratio, one of the few such verifications in the corpus [62].

A chlorophyll-based film achieved comparable functions through spatial partitioning of its components (Figure 7b). The film contained chlorophyll whose central magnesium the authors replaced with copper, together with Co and Si ions distributed in a gradient [49]. Co ions at the BiVO_4_/chlorophyll interface promoted hole transport, whereas Si ions at the outer surface increased hydrophilicity [49]. The hybrid photoanode delivered 5.1 mA cm^−2^ at 1.23 V vs. RHE in 0.5 M Na_2_SO_4_, shifted the onset potential cathodically by 350 mV, and suppressed vanadium loss [49]. The graded design constitutes an early example of function partitioning within a single molecular film, a concept developed further in Section 7.3.

Molecular bridges address a different problem: poor contact between the absorber and an inorganic cocatalyst (Figure 7c). Caffeic acid served as a bridging reagent between Fe-doped BiVO_4_ and NiFeOOH and guided a uniform self-assembled distribution of the cocatalyst [19]. The NiFe/CA/Fe-BiVO_4_ photoanode delivered 6.2 mA cm^−2^ at 1.23 V vs. RHE with a maximum ABPE of 2.15% at 0.65 V vs. RHE, and it remained stable at 0.8 V vs. RHE [19]. The plant molecule acted as a template that controlled catalyst distribution rather than as the catalytic center, a templating role that the sensitizer paradigm cannot describe.

Tannic acid-derived hole-extraction layers also combine with bulk-side engineering. Growth of NiOOH–FeOOH in the presence of tannic acid on Mo- and W-codoped BiVO_4_ coupled to CoFe_2_O_4_ raised the photocurrent from 0.96 to 6.03 mA cm^−2^ at 1.23 V vs. RHE and the charge separation efficiency to 90.2% [63]. A dual layer of a NiFe–tannic acid complex and Co(OH)_2_ raised the photocurrent of BiVO_4_ from 1.34 to 4.97 mA cm^−2^ [64]. Molecular hole-extraction layers complement doping, heterojunctions, and inorganic cocatalysts instead of substituting for them.

Comparison of the three cases exposes a benchmarking problem (Figure 7d,e). Absolute photocurrent depends strongly on the underlying BiVO_4_, which differed among the studies in morphology and doping. Enhancement factors relative to the bare electrode, combined with charge separation and injection efficiencies, allow fairer comparison than absolute photocurrent alone (Section 5.4).

Kinetic competition defines the operating window of a mediator. On bare metal oxide photoanodes, water oxidation proceeds on millisecond-to-second timescales and competes with recombination of accumulated surface holes. A molecular mediator improves performance only if it extracts holes faster than recombination occurs and passes them to the catalytic site faster than it degrades. The rates have rarely been quantified for plant-derived mediators, which leaves the kinetic basis of the reported enhancements unresolved (Section 5.2).

The bridging concept mirrors the tyrosine relay in PSII, where a redox-active residue couples the photooxidized reaction center to the Mn_4_CaO_5_ catalytic cluster. Plant molecules with catechol or carboxylate termini can play an analogous role between oxide absorbers and oxyhydroxide catalysts.

### 4.3. Coordination-Assembled Overlayers for Catalysis and Protection

Metal–phenolic networks form rapidly upon mixing polyphenols with metal salts and deposit conformally on substrates of arbitrary shape [43]. The approach requires neither vacuum equipment nor high-temperature treatment, which suits large-area photoelectrodes. The networks disassemble under strongly acidic conditions [43], so near-neutral or alkaline electrolytes are generally preferred, although acid tolerance depends on the ligand and the coordinating metal. A uniform NiFe–phenolic network coating on Mo-doped BiVO_4_ improved both water oxidation activity and stability [17], and a quercetin–Ni overlayer on WO_3_ delivered high-efficiency water splitting at pH 7 and in acid, reaching 3.23 mA cm^−2^ under acidic conditions [18]. Component controls in the same study isolate the role of the intact complex. Neither quercetin nor the nickel precursor alone formed an overlayer on WO_3_, and a carbon–nickel analogue with the organic component carbonized lowered the photocurrent to 0.38 mA cm^−2^, against 1.01 for bare WO_3_ and 2.77 for the intact complex at pH 7 [18]. Charge-transfer efficiency followed the same order, 49%, 52%, and 86% [18]. These controls support a contribution from the intact coordination architecture, but do not quantify the fraction of photocurrent attributable to quercetin alone. More generally, enhancement relative to a bare host represents the response of the complete hybrid electrode and cannot isolate the molecular contribution without appropriately matched controls.

**Figure 8:**
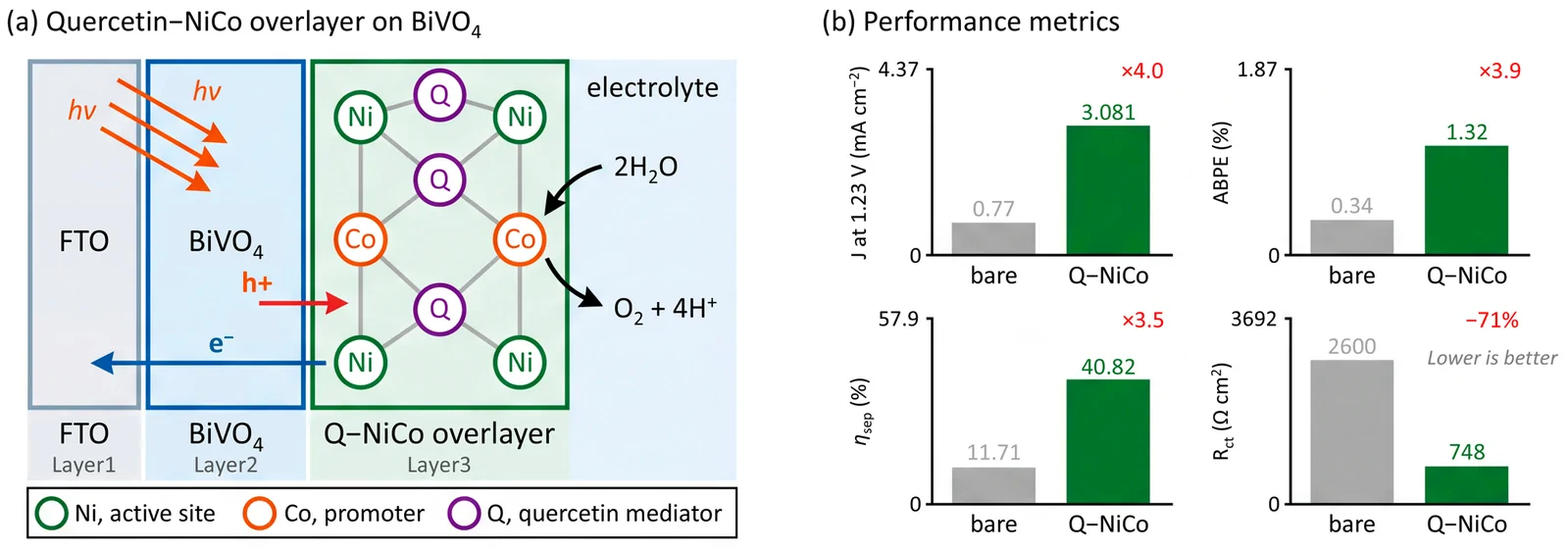
Quercetin-NiCo coordination overlayer on BiVO_4_ [20]. (**a**) Proposed component roles, with electrons and holes labeled explicitly. The Ni-active-site and Co-promoter assignments follow the computational interpretation of the source. (**b**) Photocurrent at 1.23 V vs. RHE, maximum ABPE, charge separation efficiency at 1.23 V vs. RHE, and charge-transfer resistance. Charge-transfer resistance is given in area-normalized units (Ω cm^2^).

Figure 8 presents a quercetin-coordinated NiCo overlayer (Q–NiCo) as a detailed case study. Nanoporous BiVO_4_ was obtained by electrodeposition of BiOI*_x_* and conversion with a vanadium precursor, and the Q–NiCo complex, prepared at pH 7.5, was drop-cast onto the electrode (Figure 8a) [20]. Transmission electron microscopy revealed a conformal, predominantly amorphous overlayer with a coherent interface to crystalline BiVO_4_, and elemental maps showed uniform Ni and Co dispersion without phase segregation. Photoelectron spectra showed coexisting Ni^2+^/Ni^3+^ and Co^2+^/Co^3+^ redox couples, and a weak diffraction feature suggested minor spinel-type Ni–Co oxide domains within the layer.

The overlayer lifted the photocurrent at 1.23 V vs. RHE from 0.77 to 3.081 mA cm^−2^, shifted the onset potential cathodically by approximately 100 mV, and increased the maximum ABPE from 0.34% to 1.32% (Figure 8b) [20]. The charge-transfer resistance fell from 2600 to 748 Ω cm^2^. During chronoamperometry at 1.23 V vs. RHE, the modified photoanode retained approximately 100% of its photocurrent over 24 h and 86.2% after 48 h, whereas bare BiVO_4_ retained 82.5% after 24 h [20]. The 86.2% is referenced to the day-1 value. The electrolyte was replenished after the first day, and microscopy after 48 h revealed thinning of the overlayer, which the authors linked to the subsequent decay.

DFT rationalized the division of labor among the components [20]. The Ni site exhibited balanced adsorption of oxygen evolution reaction (OER) intermediates and a minimum overpotential of 0.46 V [20]. The Co site bound *OH almost spontaneously (ΔG_1_ = −0.007 eV) and faced a strongly uphill free-energy step for O_2_ release, giving a theoretical overpotential of 0.79 V. Co therefore acted as an electronic promoter rather than a catalytic center, while quercetin mediated charge redistribution between the metal sites and the oxide. The calculations supported differentiated roles for the two coordinated metals. Experimental validation of the active sites under operating conditions remains necessary.

Ligand-based charge transport adds a further function. An ultrathin tannic acid–Fe/Co layer on BiVO_4_ delivered 4.97 mA cm^−2^ at 1.23 V vs. RHE with charge separation and injection efficiencies of 82.0% and 78.9%, and spectroscopic and computational analysis identified ligand-to-metal charge transfer as the transport pathway across the layer [65]. Tannic acid has also served to etch a Co-based zeolitic imidazolate framework, which generated coordinatively unsaturated Co sites and raised the photocurrent on BiVO_4_ from 1.38 to 4.21 mA cm^−2^ in 0.5 M potassium borate at pH 9.5 [66]. The ligand was not consumed in the process: infrared and Raman spectra of the etched framework retained the tannic acid bands, and the C 1s spectrum of the finished photoanode showed a carbonyl component at 288.4 eV assigned to tannic acid chelated to Co^2+^ [66]. The molecule therefore acted as an etchant and remained as a coordinated modifier that raised the electron density at the unsaturated Co sites, which places the study inside the inclusion criterion rather than among the cases where the precursor is consumed. An ellagic acid–mediated Co/Fe heterobimetallic complex bonded to BiVO_4_ lifted the photocurrent of BiVO_4_ from 1.55 to 5.24 mA cm^−2^ at 1.23 V vs. RHE, with 81.8% of the carriers separated and 90.2% injected. The ellagic acid result extends interfacial coordination beyond tannic acid to other oxygen-donor ligands [67]. Across these systems, polyphenols supported charge transfer, catalyst assembly, and protection. The contributions must be established separately for each architecture.

Coordination overlayers transfer across hosts. A tannin–nickel–iron complex coating promoted the activity and stability of WO_3_/BiVO_4_ heterojunctions [68], coordinated tannic acid with ternary metals advanced F-doped hematite photoanodes [69], and a tannic acid–Fe complex with dual function modified TiO_2_ nanorod arrays [70]. In supporting synthesis evidence, caffeic acid assisted solvothermal growth of an Fe-based metal–organic framework on Mo-doped BiVO_4_ [71]. That example is excluded from the molecular corpus because a retained functional molecular component was not established in the screened record. Host-dependent energy alignment, surface chemistry, and electrolyte stability govern the choice between coordination layers and sensitizers.

Relative to electrodeposited oxyhydroxide cocatalysts such as CoPi or NiFeOOH, coordination overlayers are deposited conformally from solution at ambient temperature, allow metal spacing and oxidation state to be controlled at the molecular level, and passivate the surface at the same time. The organic ligand also introduces liabilities, including oxidative degradation, acid-induced disassembly, and restructuring of metal centers under anodic bias. Post-operation spectroscopy of the Q–NiCo layer detected small positive binding-energy shifts in Ni and Co, which the authors attributed to partial surface reconstruction toward oxyhydroxide-like species [20]. Whether the active phase remains molecular or evolves into an inorganic catalyst supported by residual ligand therefore constitutes a central open question.

Coordination overlayers integrate catalysis and protection within a single phase, as also reported for NiFe–phenolic coatings [17]. The observed thinning shows that the protective function is finite, and Section 7.1 discusses strategies for regenerating the network.

### 4.4. Multicomponent Architectures: Co-Sensitization, Dyads, and Nanomaterial Hybrids

Combining complementary molecules broadens absorption and distributes functions. Such hybridization parallels established practice in colloidal quantum dot architectures, where surface ligands, capping layers and the surrounding matrix govern carrier separation and non-radiative trapping. Lead sulfide nanostructures are the archetypal case, with optical and electronic behavior set as much by surface chemistry as by particle size [72]. A semi-synthetic chlorophyll–carotenoid dyad delivered 9147 μmol g^−1^ h^−1^ of hydrogen with a turnover number of 425, about three times the rate of the chlorophyll unit alone [40]. Calculations placed the highest occupied orbital on the carotenoid and the lowest unoccupied orbital on the chlorin, so injection from the chlorin leaves the hole on the polyene and spatial separation suppresses recombination [40]. Illumination beyond 600 nm, where the carotenoid does not absorb, still yielded 1902 μmol g^−1^ h^−1^, which identifies electron donation rather than light harvesting as the dominant contribution of the carotenoid [40].

Non-covalent assembly reproduces the same function. Sequential coating of chlorin-modified Pt/TiO_2_ with β-carotene raised hydrogen evolution from 28.1 to 41.7 mmol g^−1^ h^−1^ at an equimolar ratio, whereas excess carotenoid lowered the rate by screening the chlorin from incident light [38]. The carotenoid neutralized the chlorin radical cation and blocked reverse electron transfer, a redox-spacer role distinct from sensitization [38]. Co-sensitization of betanin with lawsone, combined with Ag nanoparticles of bimodal size matched to each pigment, raised the efficiency of a natural-dye cell from 0.79% to 1.02% [46]. The three cases define a design rule: the second molecule should regenerate the first, not merely absorb additional photons. Rates measured on suspensions are reported here for the mechanistic distinction they establish, and are not comparable with the photoelectrode metrics compiled in Section 5.4.

Multicomponent assemblies require nanometer-scale control over distances. Förster energy transfer between chromophores operates over several nanometers, whereas electron transfer decays exponentially within about one nanometer of donor–acceptor separation. Plasmonic near fields likewise decay within roughly 10 nm of a metal nanoparticle surface. Layer thickness and spacer design therefore determine whether a component funnels energy, relays charge, or quenches excitation, which favors deposition methods that immobilize molecules at defined positions.

**Figure 9:**
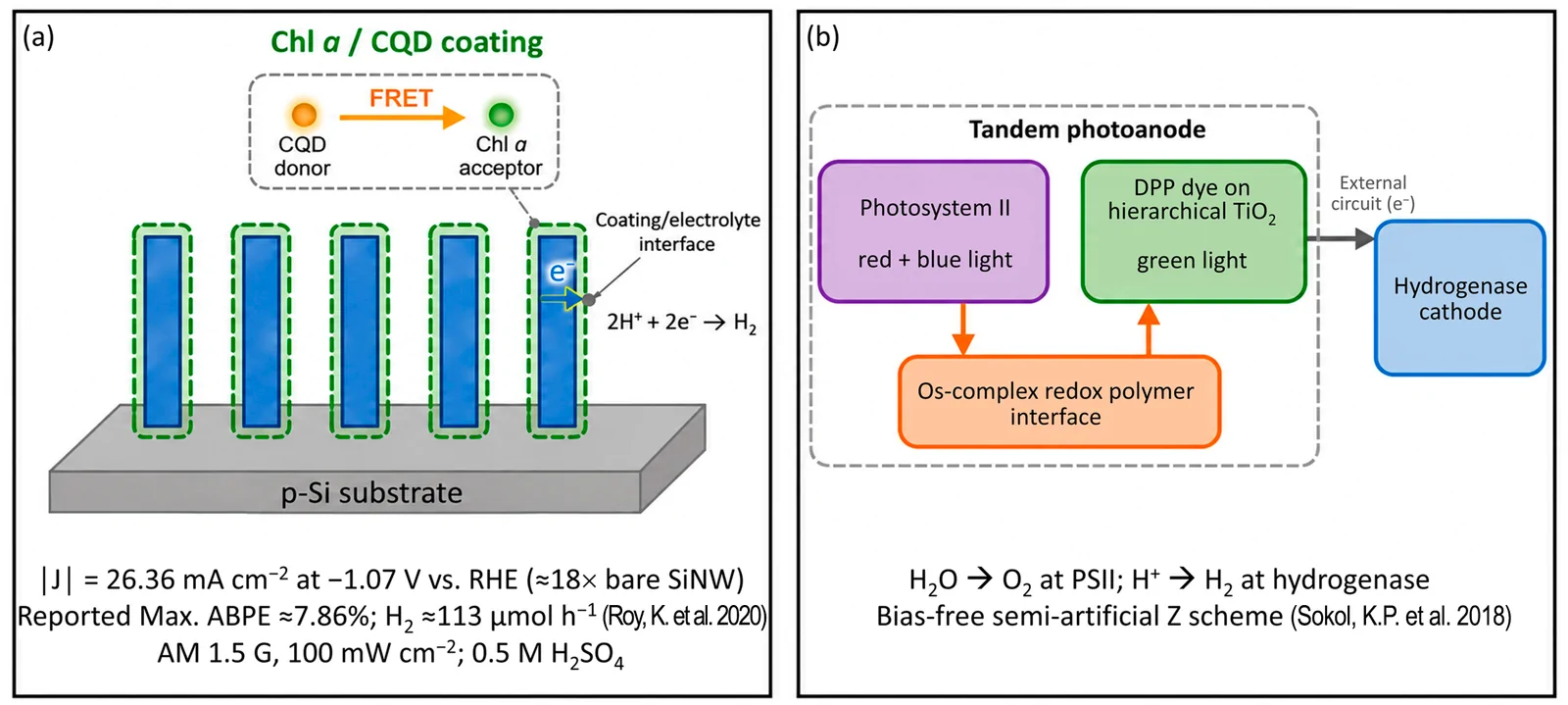
Multicomponent architectures. (**a**) Chlorophyll a/CQD-modified Si nanowire photocathode [73]. The photocurrent magnitude of 26.36 mA cm^−2^ at −1.07 V vs. RHE, the maximum ABPE of approximately 7.86%, and the hydrogen evolution rate of approximately 113 μmol h^−1^ are the values reported in the source. (**b**) Functional components of a bias-free semi-artificial device containing PSII, a dye-sensitized TiO_2_ photoanode, and a hydrogenase cathode [15]. Connecting lines identify device coupling. The biased half-cell metrics in panel (**a**) are distinct from unassisted-device efficiency.

Nanomaterial partners supply conductivity and additional interfaces (Section 3.2). On the cathodic side, a chlorophyll a/carbon quantum dot (CQD) hybrid decorated Si nanowires and increased the photocurrent magnitude to 26.36 mA cm^−2^ at −1.07 V vs. RHE, approximately eighteen times that of bare nanowires (Figure 9a) [73]. The source also reported hydrogen evolution of approximately 113 μmol h^−1^. Förster resonance energy transfer from CQDs to chlorophyll and protection of the Si surface were proposed to contribute to the enhanced response [73].

Semi-artificial photosynthesis offers a design reference for such architectures (Figure 9b). A tandem cell combined PSII, which absorbs red and blue light, with a green-absorbing diketopyrrolopyrrole dye on hierarchically structured TiO_2_ [15]. An osmium-complex-modified redox polymer mediated electron transfer between the enzyme and the electrode, and wiring of the photoanode to a hydrogenase cathode enabled bias-free overall water splitting [15]. Architectures built from small plant molecules could reproduce such spectral complementarity without relying on fragile protein assemblies.

### 4.5. Host-Specific Design Rules for Photoanodes and Photocathodes

Optimal molecular function depends on the host semiconductor (Table 4). Morphology control, cocatalyst loading and deliberate heterojunction design address recombination and photocorrosion in metal sulfide suspensions [74]. The strategies inform photoelectrode design, although suspension performance is not comparable with the photoelectrode metrics compiled here. Wide-bandgap oxides such as TiO_2_ and ZnO benefit primarily from sensitization, which requires carboxylate or catecholate anchors and an excited-state oxidation potential more negative than the conduction-band potential. Visible-light absorbers such as BiVO_4_, α-Fe_2_O_3_, and WO_3_ gain most from hole-transfer mediation, catalytic overlayers, and passivation. BiVO_4_ accounts for most reported hybrids with plant molecules [7,19,20,49].

Host chemistry constrains the electrolyte window. BiVO_4_ suffers from vanadium dissolution, and electrolyte composition strongly affects its photostability [50]; near-neutral conditions also favor polyphenol networks. α-Fe_2_O_3_ requires alkaline media in which carboxylate anchors desorb, pointing to crosslinked coordination networks as the preferred architecture. WO_3_ tolerates acidic and near-neutral electrolytes (Section 4.3). Under acidic operation, chlorophyll demetalation becomes critical and metallated chlorins are preferable.

Local pH at the electrode surface can deviate substantially from the bulk value. Water oxidation releases protons, and at current densities of several mA cm^−2^ in weakly buffered electrolytes the interfacial pH can fall markedly. Coordination networks that are stable in the bulk electrolyte may disassemble locally, so buffer capacity should be reported and optimized alongside bulk pH.

Photocathodes remain the least explored hosts, with a single study in the screened corpus [73]. Si, Cu_2_O, and NiO require molecular layers that withstand reducing conditions and transfer electrons to hydrogen evolution sites. The chlorophyll/CQD composite on Si nanowires demonstrated the feasibility of the route [73]. A chlorophyll/Cu_2_O electrode has also been reported, although the source operated it anodically [60]. Quinones, with reversible two-electron chemistry, merit investigation as electron relays on p-type photocathodes.

## 5. Mechanistic Validation and Quantitative Performance Assessment

**Figure 10:**
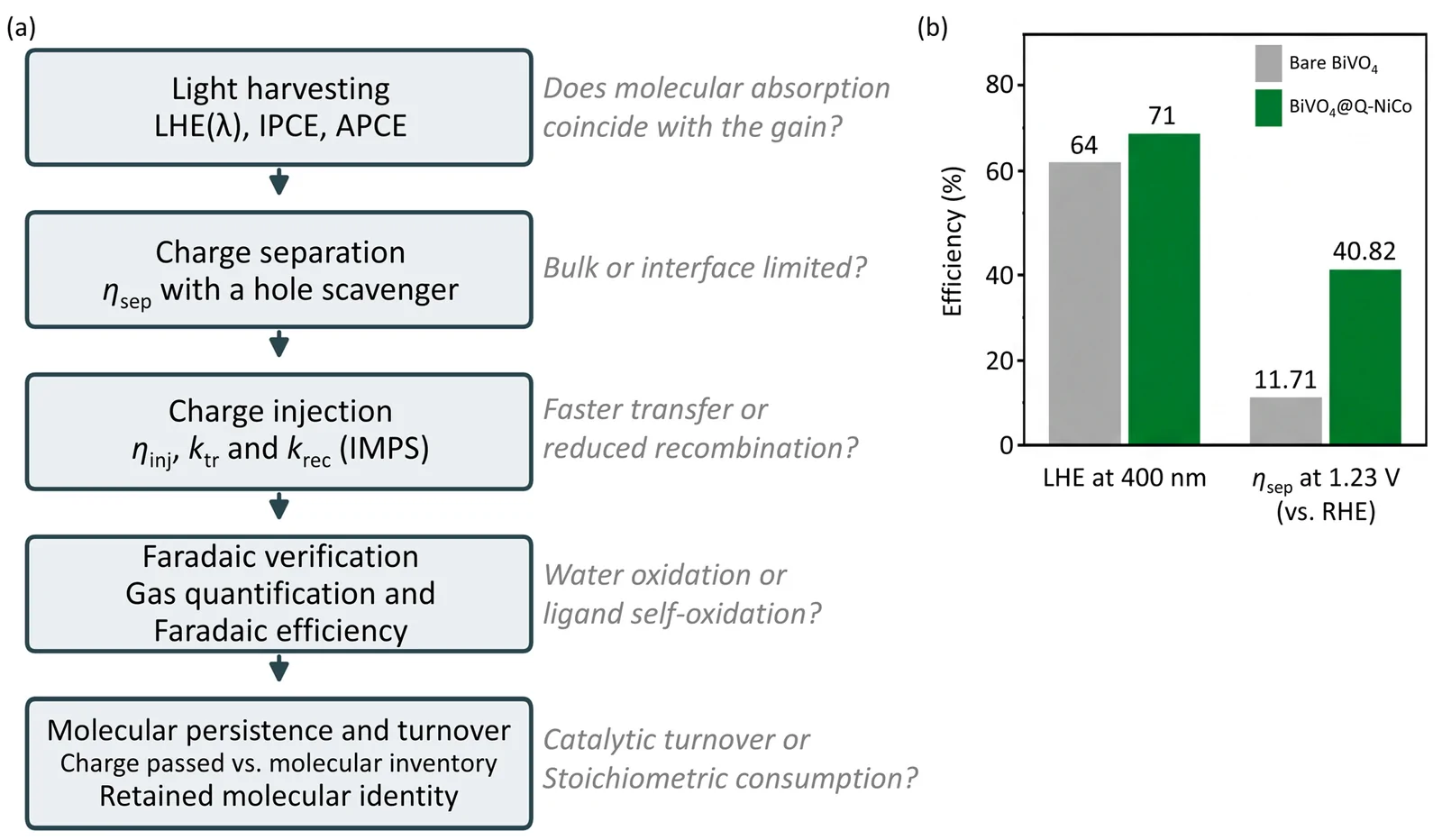
Validation workflow for molecularly modified photoanodes. (**a**) Optical, kinetic, product, and operating-state measurements required for attribution. A large accumulated charge does not independently prove intact-molecule catalysis. (**b**) Source-reported LHE at 400 nm and charge separation efficiency at 1.23 V vs. RHE for BiVO_4_ and BiVO_4_@Q-NiCo [20]. The single-wavelength LHE comparison does not quantify broadband absorption gains.

Performance gains from molecular layers can arise from several overlapping effects, including enhanced absorption, improved charge separation, faster surface kinetics, and sacrificial oxidation of the layer itself. A rigorous protocol must separate these contributions before a mechanism is assigned. Figure 10 outlines the protocol proposed here, which proceeds from optical to electronic to chemical verification. Each step answers one question and determines whether the next step is needed: whether absorption by the molecule coincides with the spectral region of the gain, whether the improvement originates in the bulk or at the interface, whether the layer mediates or passivates charge transfer, whether the current produces oxygen and hydrogen, and whether the molecule turns over catalytically. Panel (b) shows the light harvesting and charge separation measurements that implement the first two steps for a representative system.

### 5.1. Decoupling Light Harvesting, Charge Separation, and Charge Injection

The water oxidation photocurrent of a photoanode can be decomposed according to Equation (1), where J_abs_ is the photocurrent equivalent of absorbed photons, η_sep_ the charge separation efficiency, and η_inj_ the charge injection efficiency. Hole scavengers such as H_2_O_2_ or Na_2_SO_3_ enable near-unity injection and thereby isolate η_sep_, after which Equation (2) yields η_inj_ [75]. Applied to Q–NiCo-functionalized BiVO_4_, the method revealed a 3.5-fold increase in charge separation efficiency, from 11.71% to 40.82% at 1.23 V vs. RHE, whereas light harvesting efficiency (LHE) near 400 nm rose only from approximately 64% to 71% (Figure 10b) [20]. The increased separation efficiency supports an electronic contribution. The LHE value at 400 nm alone does not quantify the broadband optical contribution; that comparison requires integration of wavelength-dependent absorption with the incident photon flux. Mediation and passivation require further discrimination by kinetics. Few studies report separation and injection efficiencies together, and the tannic acid–Fe/Co layer represents one of the exceptions [65].

(1) J_H2O_ = J_abs_ × η_sep_ × η_inj_

(2) η_inj_ = J_H2O_/J_scavenger_

The scavenger method assumes that the molecular layer is inert toward the scavenger and does not itself consume holes. Polyphenol and quinone layers can violate both assumptions. Control experiments on the isolated molecular layer, deposited on an inert conductive substrate, should accompany any efficiency decomposition. The reported 3.5-fold rise in separation efficiency for the drop-cast Q–NiCo overlayer carries that caveat and does not by itself locate the gain in the bulk of the host.

Spectrally resolved efficiencies discriminate sensitization from interfacial effects. The incident and absorbed photon-to-current efficiencies follow Equations (3) and (4), where λ is the wavelength in nm, J the photocurrent density in mA cm^−2^, P the incident power density in mW cm^−2^, and LHE the wavelength-dependent absorbed fraction, expressed from 0 to 1. An IPCE increase beyond the host absorption edge supports sensitization. An increase in the absorbed photon-to-current efficiency (APCE) can reflect improved charge transfer or reduced recombination and is not unique to a mediator. Spectral measurements constrain the assignment and should be combined with kinetic and chemical evidence.

(3) IPCE(λ) = 1240 × J(λ)/[λ × P(λ)] × 100%

(4) APCE(λ) = IPCE(λ)/LHE(λ)

### 5.2. Probing Interfacial Charge-Transfer Dynamics

Electrochemical impedance spectroscopy (EIS) quantifies the charge-transfer resistance at the photoanode/electrolyte interface, and Mott–Schottky analysis reports changes in donor density and flat-band potential. In Q–NiCo-functionalized BiVO_4_, the donor density increased from 4.30 × 10^19^ to 1.48 × 10^20^ cm^−3^, and the flat-band potential shifted cathodically from 0.6529 to 0.5827 V vs. RHE [20]. The open-circuit photovoltage increased from approximately 140 to 190 mV, and photovoltage decay analysis indicated prolonged carrier lifetimes. Mott–Schottky analysis assumes a planar, uniformly doped electrode and a frequency-independent depletion capacitance. A nanoporous film carrying a conformal overlayer may violate these assumptions, so extracted donor densities should be interpreted cautiously as apparent trends rather than validated absolute carrier concentrations.

Intensity-modulated photocurrent spectroscopy provides a role-specific diagnostic. The method resolves pseudo-first-order rate constants for charge transfer (k_tr_) and surface recombination (k_rec_), and η_inj_ equals k_tr_/(k_tr_ + k_rec_). A catalytic mediator primarily raises k_tr_, whereas a passivating layer primarily lowers k_rec_. Measuring both constants assigns the dominant function of a molecular layer directly and complements the role-based classification of Section 4.

Steady-state measurements do not uniquely distinguish catalytic mediation from passivation. Transient absorption and intensity-modulated photocurrent spectroscopy (IMPS) can compare hole-transfer and recombination rates, while operando vibrational spectroscopy can probe the chemical state of the layer. C–O–M and C=O signals persisted after 48 h for the quercetin–NiCo system [20], supporting retention of organic components. Those signals alone do not prove preservation of the original active coordination network. Broader use of time-resolved and operando methods is needed to assign operating mechanisms.

### 5.3. Faradaic Verification: Water Oxidation Versus Molecular Self-Oxidation

Oxidizable molecules create a specific pitfall. Catechols, galloyl units, and hydroquinones oxidize at potentials accessible to photogenerated holes and well below the water oxidation potential (Section 3.1). Photocurrent may originate partly from irreversible oxidation of the molecular layer, particularly during short measurements. Such current inflates apparent activity and simultaneously signals degradation.

Quantification of evolved O_2_ and H_2_ provides the decisive test. For Q–NiCo-functionalized BiVO_4_, gas chromatographic analysis at 10 min intervals over 120 min showed that the evolved O_2_ and H_2_ tracked the amounts calculated from the photocurrent, with FE values of approximately 90% for O_2_ and 95% for H_2_ [20]. Many reports on natural sensitizers lack comparable verification and rely solely on photocurrent.

A molecular turnover criterion is proposed here as a complementary check. The literature has conflated two tests that need to be kept apart. The first tests whether stoichiometric oxidation of the assumed molecular inventory can account for the accumulated charge: the cumulative charge passed must exceed the charge required to oxidize the entire molecular inventory. At 1 nmol cm^−2^ and two electrons per molecule, ten times that inventory corresponds to 1.93 mC cm^−2^, which is passed in approximately 1.9 s at 1 mA cm^−2^, so the test excludes only stoichiometric behavior and is a floor, not a target. The second establishes whether the layer survives, and is the durability requirement derived in Section 3.3. Where the ligand is a spectator and the metal carries the catalysis, turnover per ligand measures the survival demand on the ligand, not catalytic activity, and the two must be reported separately. A large inventory-normalized charge and high O_2_ FE constrain the contribution of stoichiometric self-oxidation. They do not exclude concurrent ligand degradation or prove that an intact molecular layer remained the active catalyst. Post-operation inventory measurements and operating-state characterization are also required. Isotope labeling with H_2_^18^O can further confirm that evolved oxygen originates from water.

The molecular inventory required for the criterion can be determined by desorption followed by UV–vis quantification, by quartz crystal microgravimetry during deposition, or by elemental analysis of metal centers. Inductively coupled plasma mass spectrometry of the electrolyte after operation additionally reports leaching of Co, Ni, or Fe from coordination layers and links activity loss to a specific degradation mode.

### 5.4. Benchmarking and Reporting Standards

Table 5 compiles representative photoanodes modified with plant-derived components, most of them BiVO_4_ photoanodes. Each entry lists the molecular component, the interfacial role assigned to it, the photocurrent density at the stated potential, available measurement conditions, and the additional metrics reported in the source, so that the same rows expose both the performance and the depth of the supporting evidence. Photocurrents on BiVO_4_ span 3.08 to 6.2 mA cm^−2^, while the single hematite entry reaches 3.3 and is not comparable with the BiVO_4_ entries. Hole-transfer, bridging and coordination entries span that whole range, and the single sensitizing entry was measured against Ag/AgCl and cannot be placed on the same scale, so the comparison indicates where the evidence is concentrated, leaving open which role performs best. Differences in electrolyte composition and pH, illumination intensity and geometry, electrode area, film thickness, and molecular or catalyst loading limit direct comparison of absolute photocurrents across studies. Table 5 therefore supports comparisons within individual studies more reliably than a ranking across studies. The reporting items proposed below address the gap. Established protocols for PEC reporting [76] should be extended with molecule-specific items.

A minimum reporting set is recommended in Table 6. The items fall into four groups: identity and loading of the molecular layer, which establish what was placed at the interface; efficiency decomposition and product quantification, which determine the origin of the measured current; inventory-normalized durability, which compares cumulative charge with the molecular inventory without equating ligand survival with catalytic turnover; and the sustainability inventory, which supplies the input data for a life-cycle comparison. A near-term target accompanies each item in the final column: 100 h of operation with 90% retention, FE of at least 95%, and at least 10^6^ electrons per initially loaded molecule at a loading of 1 nmol cm^−2^. The three studies reporting oxygen FE give approximately 90% [20], above 93% [62], and above 86% [58]. The latter two lower bounds do not establish whether the 95% target was reached; the available values therefore do not support classifying all three entries as below the target. Reporting and benchmarking then rest on the same quantities. The set comprises photocurrent density at 1.23 V vs. RHE, onset potential, ABPE, IPCE, and FE for O_2_ and H_2_. Stability data should state duration, retention, post-operation characterization, and any electrolyte replenishment, as disclosed for Q–NiCo-functionalized BiVO_4_ [20]. Molecular source, purity, and surface loading allow normalization per molecule.

## 6. Sustainability Assessment Beyond Natural Origin

Natural origin does not guarantee sustainability. The environmental benefit of a plant-derived component depends on how the molecule is sourced and purified, how long the device operates, and which additional elements accompany the molecule in the final electrode.

### 6.1. Sourcing, Extraction, and Batch-to-Batch Variability

Extraction methods for natural pigments range from simple solvent extraction to multistep purification [13]. Crude extraction is inexpensive and mild, whereas purification to structurally defined compounds multiplies solvent and energy demand. The trade-off between reproducibility and environmental burden should be quantified, not assumed.

Feedstock choice can reconcile the two goals. Agricultural and food-processing residues, such as fruit peels and spent leaves, provide pigments and polyphenols without competing with food production. Some classes face biological supply limits; betalains, for example, occur within the Caryophyllales, which restricts sources to a limited set of crops [41]. Seasonal and cultivar-dependent variation calls for standardized quality metrics, such as chromatographic purity and molar absorptivity, for each batch.

Cascading use reconciles the two routes of biomass utilization. A residue can first release its extractable pigments and polyphenols and afterward serve as a carbon precursor for electrode materials [11,12]. Sequential valorization raises the value obtained per unit of biomass and removes the competition between molecular and carbon uses of the same feedstock. Life-cycle accounting for plant-derived photoelectrodes should allocate burdens across the whole cascade rather than to the extracted molecule alone.

The mass of molecule contained in a device is small. At a loading of 1–100 nmol cm^−2^, a molecule with a molar mass near 300 g mol^−1^, such as quercetin, amounts to roughly 3–300 mg per square meter of electrode. The environmental footprint is dominated by extraction and purification solvents, accompanying metals, and replacement frequency rather than by the molecule itself. Sustainability priorities accordingly shift from feedstock abundance toward low-solvent purification and long operating lifetimes.

### 6.2. Life-Cycle, Cost, and Toxicity Considerations

A toxicity assessment framework based on the periodic table and recommended daily intake has been applied to photosensitizers used in PEC hydrogen production [14]. Plant-derived ligands generally raise little intrinsic concern. The metal centers that accompany them, including Co and Ni in coordination overlayers, require explicit evaluation of leaching and end-of-life fate.

Plant-derived denotes a native scaffold biosynthesized by plants, including derivatives that retain that scaffold; it does not certify the supply chain of a reagent. The principal systems discussed here combine organic ligands with Co, Ni, or Fe and often a vanadium-containing absorber. Their environmental burden requires evaluation of the complete electrode and processing route.

Technoeconomic analyses of solar hydrogen have highlighted the strong dependence of hydrogen cost on conversion efficiency and device lifetime [77]. An inexpensive molecular layer with a short lifetime may raise the cost of hydrogen through frequent replacement. Life-cycle assessments (LCAs) for plant-derived hybrids should adopt a functional unit per kilogram of hydrogen and include extraction, electrode fabrication, and replacement cycles. No such assessment was found for plant-derived PEC electrodes, which marks a clear priority for future work.

End-of-life behavior carries a less appreciated advantage. Metal–phenolic networks disassemble under acidic conditions [43], a liability during operation that becomes an asset at the end of service. Mild acid treatment could strip a degraded overlayer, recover the metal ions, and regenerate the underlying semiconductor for recoating. Refurbishment of this kind would extend the service life of the inorganic absorber, which dominates the embodied energy of the electrode.

## 7. Challenges and Future Perspectives

The preceding sections established where plant-derived molecules function at photoelectrodes and how their role can be verified. Four barriers now separate the field from practical devices: operational durability, the unexplored breadth of natural product chemistry, architectural integration of multiple functions, and demonstration beyond half-cell measurements.

### 7.1. Closing the Stability Gap Toward Practical Lifetimes

Reported stability for plant-derived hybrids extends from hours to about two days. Even the Q–NiCo overlayer, among the most durable examples, lost part of its photocurrent within 48 h [20]. Economically viable operation, by contrast, requires lifetimes that are orders of magnitude longer [77], corresponding to the per-molecule turnover requirement derived in Section 3.3 and the intermediate targets in Table 6.

Each degradation route suggests its own remedy. Thinning of the coordination overlayer during operation [20] points toward self-healing designs, in which dissolved ligands and metal ions in the electrolyte continuously redeposit the network, a strategy that parallels the electrolyte engineering used to stabilize BiVO_4_ [50]. Crosslinked or hydrophobic top layers and semi-synthetic anchors of higher hydrolytic stability remain to be tested. Evidence from other nanostructured systems shows that the composition of such a layer decides the outcome. A conjugated polymer shell on silver nanoparticles reached a corrosion protection efficiency of 75.5% in its neutral state, whereas chloride doping of the same shell accelerated attack [78]. Overlayer chemistry therefore requires validation alongside the molecular identity beneath it.

Accelerated stress protocols are needed to evaluate such remedies within practical timescales. Protocols should combine elevated light intensity, potential cycling that mimics diurnal start–stop operation, and elevated temperature. Reporting the temperature dependence of degradation would allow estimation of an activation energy and extrapolation of lifetimes under field conditions.

### 7.2. Exploring Natural-Product Chemical Space with Machine Learning

Current studies have examined only a handful of plant molecules, dominated by tannic acid, quercetin, caffeic acid, and chlorophyll. Open databases containing hundreds of thousands of natural products [53] have not been exploited for PEC applications. Machine learning models trained on computed frontier orbital energies, chelation motifs, and redox potentials could prioritize candidates for each interfacial role. A genetic algorithm coupled to a neural network has already selected combinations of natural leaf extracts for water splitting electrodes even when the sources were crude [56].

Autonomous experimentation could close the loop. Robotic dip-coating or drop-casting, coupled with rapid photoelectrochemical screening, would generate the consistent datasets that machine learning requires. Spectroscopic identification of unknown constituents in plant extracts would, in parallel, feed new structures into the design space. Failed molecules are equally valuable for model training and should be reported in machine-readable form, including MRL, loading, electrolyte, and verification data.

### 7.3. Photosynthesis-Inspired Multifunctional Assemblies

PSII integrates light harvesting, charge separation, donor-side hole transfer, acceptor-side electron transfer, and photoprotection. Corresponding functions motivate combinations of chlorophyll derivatives, carotenoids, redox relays, and catalytic coordination networks. An integrated small-molecule assembly remains a design objective requiring independent validation of each transfer step.

**Figure 11:**
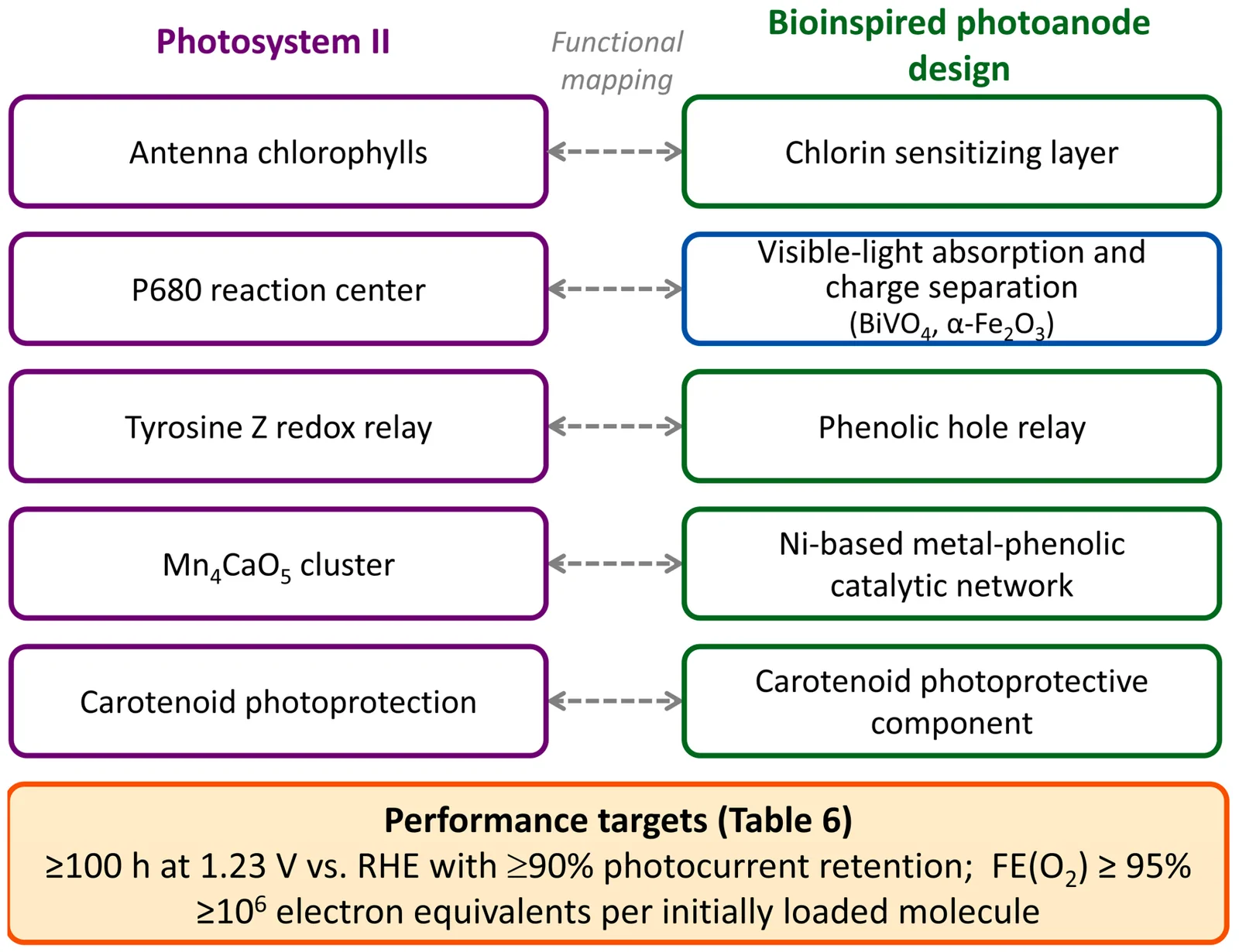
Functional mapping between PSII and a proposed molecular photoanode. Each row pairs a PSII component with the engineered component proposed to perform the analogous function, and the horizontal dashed arrows mark that correspondence. The upper rows follow the donor-side sequence, from antenna chlorophylls and the P_680_ reaction center through the tyrosine Z relay to the Mn_4_CaO_5_ cluster, whose engineered counterparts are a chlorin sensitizing layer, a visible-light absorber, a phenolic hole relay, and a nickel-based metal–phenolic catalytic network. The blue box marks the semiconductor host, which absorbs visible light and separates charge in place of the reaction center. Carotenoid photoprotection occupies the final row because it acts on every stage rather than within the transfer sequence. The lower panel states the performance targets of Table 6 against which such a design would be judged. The mapping is conceptual; a complete sensitizer-regeneration cycle and an experimentally validated relay sequence are not implied.

Figure 11 maps selected PSII functions onto proposed photoanode components. Excitation energy reaches the P_680_ reaction center, and the oxidizing equivalent is transferred through tyrosine Z to the Mn_4_CaO_5_ cluster. The Q_A_/Q_B_ quinones operate on the electron-acceptor side. In an engineered electrode, a chlorin sensitizer could inject electrons into a semiconductor, while a separate donor-side pathway transfers holes toward catalytic sites. Caffeic acid bridges provide evidence for interfacial electronic coupling [19], without proving reversible phenoxyl cycling. A quinone-based hole relay remains a hypothesis requiring suitable one-electron potentials and kinetics. Sensitizer regeneration and competing transfer pathways must also be resolved before the components form a complete reaction cycle. Semi-artificial tandem systems demonstrate functional integration with intact photosystems [15], providing a reference for small-molecule designs.

### 7.4. Integration into Unassisted Tandem Devices

Unassisted water splitting requires pairing a photoanode with a complementary photocathode or photovoltaic cell. Plant-derived hybrids have so far been evaluated almost exclusively in three-electrode half-cell configurations. Coupling a molecularly modified BiVO_4_ photoanode with a Si-based photocathode, including chlorophyll-activated Si nanowires [73], offers a straightforward first demonstration.

Unassisted solar-to-hydrogen (STH) efficiency is determined by the operating current of the complete device, product FE, and incident optical power. Photocurrents measured at 1.23 V vs. RHE in a biased half-cell cannot be converted directly into unassisted STH efficiencies. The ABPE values compiled in Table 5, 0.34 to 2.15%, were measured under applied bias and remain distinct from STH efficiency. A tandem demonstration requires current matching, an unbiased operating point, and gas quantification for the assembled device.

Replacing water oxidation with a value-added reaction relaxes the thermodynamic constraint. NiFe phenolic networks on a BiVO_4_ photoanode enabled selective valorization of glycerol to formic acid [79]. Molecular coordination layers thus transfer to anodic reactions of lower overpotential. Pairing such an anode with hydrogen evolution converts the same molecular design into a route toward hydrogen and a chemical product.

Solution processing favors scale-up. Metal–phenolic networks assemble in one step on large and irregular substrates [43], and drop-casting sufficed to deposit quercetin–NiCo overlayers [20]. Solution-phase routes to polymer nanostructures, where template and template-free strategies set particle size and morphology, offer a precedent for controlling film architecture over large areas [80]. Uniformity over large areas, operation under natural sunlight, and management of local pH gradients will determine whether molecular hybrids progress beyond laboratory electrodes.

## 8. Conclusions

Plant-derived functional molecules have progressed from low-cost substitutes for synthetic dyes to multifunctional components of photoelectrochemical interfaces. The evidence reviewed here is concentrated on hole-transfer mediation, molecular bridging, and coordination-assembled overlayers rather than on sensitization alone, and the compilation does not yet support a ranking among those roles. Hybrid layers pairing these molecules with cobalt, nickel or iron centers raised BiVO_4_ photocurrents to approximately 3–6 mA cm^−2^ at 1.23 V vs. RHE while combining catalysis with protection against photocorrosion.

Three quantitative considerations guide validation. Net two-electron phenol/quinone potentials do not determine the feasibility of a one-electron ligand relay, which requires separate measurements. At a chlorophyll loading of 0.1 nmol cm^−2^, the calculated upper bound within 640–680 nm is approximately 0.05 mA cm^−2^. Additional absorption bands must be assessed before attributing broadband gains to interfacial effects. Finally, 1000 h at 1 mA cm^−2^ and 1 nmol cm^−2^ corresponds to approximately 3.7 × 10^7^ electrons per initially loaded molecule. For spectator ligands, that number expresses a survival requirement rather than catalytic turnover.

Further progress depends on three methodological advances. Structurally defined molecules must replace crude extracts to establish structure–function relationships, for which the readiness scale of Table 3 provides a reporting standard. Faradaic verification and molecular turnover criteria must exclude self-oxidation as a source of photocurrent. Sustainability must be demonstrated through life-cycle and toxicity assessment rather than inferred from natural origin.

A further obstacle deserves explicit statement. Most of the natural product inventory remains structurally uncharacterized, and plant extracts contain closely related congeners that differ by a hydroxyl group, a glycoside, or a single double-bond configuration, yet differ substantially in redox potential, binding strength, and photostability. Isolation at analytical purity is slow, reference spectra are often unavailable for minor constituents, and the amounts recovered are frequently too small for conventional structure elucidation. Studies therefore default to crude extracts, which returns the field to the reproducibility problem identified in Section 2.1.

Section 3.4 assembles the tools for a way through. Multimodal spectral fingerprints combined with computed spectra can assign constituents without isolation at preparative scale, dimensionality reduction groups congeners by structural similarity, and curated natural product databases narrow the candidate assignments before any synthesis is attempted. Quantum chemical descriptors then rank the assigned structures for a specific interfacial role, and autonomous measurement loops test the shortlist under the verification protocol of Section 5. Identification, prediction, and verification stop being separate activities.

The role-based framework we propose here links molecular chemistry to interfacial function and device metrics. Coupled with data-driven exploration of natural product space and photosynthesis-inspired assembly, the framework provides a roadmap toward durable and efficient photoelectrodes for solar hydrogen production. Whether a plant-derived route is also the more sustainable one remains to be established by life-cycle comparison against the synthetic alternative, for which Table 6 specifies the inventory items to report.

## Figures and Tables

**Table 3:** MRLs for plant-derived components in photoelectrochemical studies. LC–MS, liquid chromatography–mass spectrometry; NMR, nuclear magnetic resonance.

MRL	Material	Minimum Reported Characterization	Supported Conclusions
1	Crude extract	Source species, plant tissue, solvent, and extraction conditions	Proof of concept
2	Enriched fraction	Class-level assay, such as total anthocyanin or polyphenol content	Class-level trends
3	Profiled mixture (reported identities)	Major constituents named; analytical confirmation and relative composition recorded where reported	Mixture-specific trends; constituent-specific attribution requires quantitative profiling
4	Defined single compound	Compound named with its source; purity recorded where reported	Molecule-specific attribution; mechanism requires independent chemical and kinetic evidence
5	Engineered derivative	Full characterization of the modified anchor, metal center, or substituent	Transferable design rules

**Table 4:** Host-specific design rules for plant-derived molecular components.

Host	Primary Limitation	Preferred Molecular Role	Recommended Molecular Features	Electrolyte Window	Status
TiO_2_, ZnO (n-type)	Absorption limited to ultraviolet light	Photosensitization	Carboxylate or catecholate anchors; excited-state oxidation potential more negative than host conduction-band potential	Near-neutral (ZnO dissolves in acidic and strongly alkaline media)	Demonstrated
BiVO_4_ (n-type)	Surface recombination; slow oxygen evolution; vanadium dissolution	Hole-transfer mediation; coordination overlayers	Catechol or galloyl ligands coordinated to Ni, Co, or Fe centers	Near-neutral	Demonstrated
α-Fe_2_O_3_ (n-type)	Short hole diffusion length; surface states	Passivation; catalytic overlayers	Ultrathin crosslinked coordination networks	Alkaline	Demonstrated
WO_3_ (n-type)	Limited visible absorption	Catalytic coordination overlayer; charge-transfer promotion	Flavonoid–Ni overlayers; quinones	Acidic to neutral	QNi/WO_3_ overlayer demonstrated [18]; quinone relay proposed
Si, Cu_2_O, NiO (p-type)	Surface oxidation (Si); photocorrosion (Cu_2_O)	Electron relay; sensitization (NiO)	Reductively stable chromophores; quinone relays	Acidic to near-neutral	Si example demonstrated; Cu_2_O, NiO, and quinone-relay designs proposed

**Table 5:** Representative PEC performance and available measurement conditions of photoanodes modified with plant-derived molecules.

Photoanode	Molecule	Primary Role	J	Bare Host	Available Measurement Conditions	Other Metrics	Stability; FE	Ref.
BiVO_4_/TA–Co	Tannic acid	Hole transfer; cocatalyst	4.8	1.45	n.r.	~3-fold; maximum ABPE 0.96% at 0.74 V	n.r.	[7]
BiVO_4_/Chl–Co–Si film	Copper-substituted chlorophyll	Hole transport; passivation	5.1	n.r.	0.5 M Na_2_SO_4_, neutral; AM 1.5G, 100 mW cm^−2^	Onset shifted cathodically	n.r.	[49]
NiFe/CA/Fe-BiVO_4_	Caffeic acid	Molecular bridge to NiFeOOH	6.2	n.r.	n.r.	Maximum ABPE 2.15% at 0.65 V	Stable at 0.8 V (qual.); FE n.r.	[19]
BiVO_4_@Q–NiCo	Quercetin	Coordination overlayer	3.081	0.77	0.5 M Na_2_SO_4_, pH 7.3	~4-fold; ABPE 1.32%; η_sep_ 40.8%	48 h; 86.2% ^1^; FE 90% O_2_, 95% H_2_	[20]
BiVO_4_/TAFC	Tannic acid	Ligand-to-metal charge transfer	4.97	n.r.	n.r.	η_sep_ 82.0%; η_inj_ 78.9%	n.r.	[65]
BiVO_4_/NFTA/Co	Tannic acid	Dual hole-extraction layer	4.97	1.34	n.r.	3.7-fold	n.r.	[64]
TA@ZIF-L(Co)/BiVO_4_	Tannic acid	Etchant and retained Co modifier	4.21	1.38	0.5 M KBi, pH 9.5	3-fold; ABPE 1.33% at 0.67 V; η_inj_ 80%	3 h; 87%; FE n.r.	[66]
MW:BVO@CFO/TANF	Tannic acid	Hole-extraction layer	6.03	0.96	n.r.	η_sep_ 90.2%	n.r.	[63]
BiVO_4_/Co-EA/FeOOH	Ellagic acid	Hole transport layer; interfacial bonding	5.24	1.55	0.5 M KBi	3.4-fold; ABPE 2.02%; η_sep_ 81.8%; η_inj_ 90.2%	9 h; stable (qual.); FE n.r.	[67]
Ti-Fe_2_O_3_/GA/TACo	Gallic acid, tannic acid	Bridging ligand; surface passivation	3.3	0.33	1 M KOH	10-fold; ABPE 0.34%; η_inj_ 75%; IPCE 78% at 390 nm	5 h, 87%; FE(O_2_) > 93%	[62]
ChlCuNa–BiVO_4_	Cu-chlorophyllin	Sensitization	n.c.	n.c.	AM 1.5, 100 mW cm^−2^; transient photocurrent at 1.6 V vs. Ag/AgCl; electrolyte n.r.	1.82-fold at 1.6 V vs. Ag/AgCl; H_2_ rate 2.06 → 5.43 μmol h^−1^ cm^−2^	4 h; little decay (qual.); FE > 86% for O_2_ and H_2_	[58]

Notes: Photocurrent densities (J) are given in mA cm^−2^ at 1.23 V vs. RHE, except where marked otherwise. The measurement-conditions column lists information verified in the cited sources and is not intended as a complete experimental specification. Omitted conditions are not assumed to be identical across studies. System labels follow the source publications: TA, tannic acid; CA, caffeic acid; GA, gallic acid; EA, ellagic acid; TACo, tannin-cobalt; TAFC, tannic acid-Fe/Co; TANF, tannic acid-NiFe; NFTA, NiFe-tannic acid; Chl, chlorophyll; ChlCuNa, sodium copper chlorophyllin; MW:BVO, Mo, W-codoped BiVO_4_; CFO, CoFe_2_O_4_. Entries marked n.r. were not located in the source, and n.c. indicates a value that is not comparable because it was measured on a different potential scale. The n.r. designation records a retrieval limitation and does not establish that the measurement was never performed. Q, quercetin; KBi, potassium borate buffer; ZIF-L, zeolitic imidazolate framework with a layered structure. Qualitative stability descriptions are not numerical retention values. Performance entries include Ref. [20] from 2026, outside the bibliometric window. ^1^ The 86.2% retention is relative to the day-1 current; the electrolyte was replenished after 24 h. Qual. indicates a qualitative stability description. The identical value of 4.97 mA cm^−2^ for BiVO_4_/TAFC and BiVO_4_/NFTA/Co was verified against both sources and is coincidental. The primary-role column states the function reported in each source; the four-role classification used for the counts in Figure 1e and Table S3 is defined in Section 4. Molecular readiness levels (Table 3) for the compiled entries are MRL 4, except the Chl–Co–Si film at MRL 3; Ref. [20] is also assigned MRL 4 from the reported quercetin identity but lies outside the 2005–2025 screening window and is excluded from the distribution in Figure 1f and Table S3.

**Table 6:** Proposed minimum reporting items and near-term targets for plant-derived hybrid photoelectrodes.

Category	Required Items	Rationale	Proposed Near-Term Target
Molecular identity	MRL (Table 3), source, purity, surface loading	Attribution and per-molecule normalization	MRL of at least 4
Activity	J at 1.23 V vs. RHE, onset potential, ABPE, IPCE and APCE; electrolyte composition, bulk pH, buffer identity and concentration, and buffer capacity where available	Separation of sensitization from interfacial effects	Enhancement factor and APCE reported relative to the bare host
Mechanism	η_sep_, η_inj_, k_tr_, and k_rec_	Assignment of the interfacial role	Consistent role assignment from at least two independent methods
Product verification	FE for O_2_ and H_2_; cumulative charge versus molecular inventory	Exclusion of ligand self-oxidation	FE(O_2_) of at least 95%; inventory-normalized electron count ≥10^6^. For spectator ligands, this is a durability exposure metric, not a catalytic turnover number.
Durability	Duration, current retention, electrolyte replenishment or exchange, post-operation spectroscopy, and metal leaching	Identification of degradation modes	At least 100 h at 1.23 V vs. RHE with 90% retention and no electrolyte replenishment
Sustainability	Solvent and energy use for extraction and purification, metal content, replacement interval	Input data for life-cycle assessment	Inventory reported per square meter of electrode

## Data Availability

No new data were created in this review. The Scopus query strings and document counts used for Figure 1 are listed in Table S1.

## Supplementary Materials

The following supporting information can be downloaded at: https://www.mdpi.com/article/10.3390/ijms27209326/s1.
