# Supplementary material for: Plant-Derived Functional Molecules for Photoelectrochemical Water Splitting: From Molecular Design to Hybrid Materials Engineering
Source: Int J Mol Sci. 2026 Oct 20;27(20):9326. doi: 10.3390/ijms27209326 (PMC13705525; doi:10.3390/ijms27209326)
Supplement: Supplementary file 1 [file ijms-27-09326-s001.zip › ijms-4621629-Supporting_Information.pdf]

Supporting Information

Plant-Derived Functional Molecules for Photoelectrochemical Water Splitting: From Molecular Design to Hybrid Materials Engineering

Sangmin Kim [1,†], Inseo Hwang [1,†], Gihoon Kim [2], and Hyeonseok Yoon [1,2,*]

1 Department of Polymer Engineering, Graduate School, Chonnam National University, 77 Yongbong-ro, Gwangju 61186, Republic of Korea
2 School of Polymer Science and Engineering, Chonnam National University, 77 Yongbong-ro, Gwangju 61186, Republic of Korea
* Correspondence: hyoon@chonnam.ac.kr

† These authors contributed equally to this work.

Table S1, Scopus query strings and document counts; Table S2, screening flow; Table S3, the 26 retained studies with molecular component, primary interfacial role, and molecular readiness level; Table S4, the 33 excluded records and exclusion reasons. An optical upper-bound calculation and Table S5 on energy-value provenance follow Tables S1–S4. Figures in the main text are schematics or plots of cited numerical data.

Table S1. Scopus query strings and document counts (primary search 21 September 2026; supplementary search 8 October 2026).

Dataset	Query string	Documents
PEC water splitting	TITLE-ABS-KEY (photoelectrochemical AND ("water splitting" OR "water oxidation" OR "oxygen evolution" OR "hydrogen evolution")) AND PUBYEAR > 2004 AND PUBYEAR < 2026	13,271
PEC reviews	As above, AND DOCTYPE (re)	879
PEC with plant-derived molecules (primary)	As above, AND ("natural dye*" OR "natural pigment*" OR "natural sensiti*" OR "plant-derived" OR "plant extract*" OR chlorophyll* OR "chlorin e6" OR carotenoid* OR "beta-carotene" OR betalain* OR betanin OR anthocyanin* OR polyphenol* OR "tannic acid" OR "metal-phenolic" OR flavonoid* OR quercetin OR "caffeic acid" OR lawsone OR juglone OR alizarin)	45
Supplementary search on omitted terms	TITLE-ABS-KEY ((photoelectrochemical OR photoelectrocatalytic OR photoanode) AND ("water splitting" OR "water oxidation" OR "oxygen evolution" OR "hydrogen evolution") AND (tannin* OR "gallic acid" OR catechol* OR "ellagic acid")) AND PUBYEAR > 2004 AND PUBYEAR < 2026	22 (15 new)
DSSCs with natural dyes	TITLE-ABS-KEY (("dye-sensitized solar cell*" OR DSSC) AND ("natural dye*" OR "natural pigment*" OR chlorophyll* OR carotenoid* OR betalain* OR anthocyanin* OR flavonoid*)) AND PUBYEAR > 2004 AND PUBYEAR < 2026	1607

The supplementary query returned 22 records, including 15 new records after removal of seven duplicates. Three new records met the inclusion criteria: the gallic acid/tannic acid system, the ellagic acid system, and the TiO_2–catechol study. The final corpus comprises 23 primary-query studies and three supplementary-query studies. The tannic-acid-derived carbon study of Choi et al. was excluded because the molecular precursor was consumed during carbonization. The 0.34% ratio in the main text refers only to the primary query.

Table S2. Flow of records through screening.

Stage or category	Records	Listed in
Retrieved by the primary query	45	—
Retrieved by the supplementary query, after removing 7 duplicates	15	—
Total screened on title, abstract and full text	60	—
Retained: molecule acts at the electrode interface during PEC water splitting	26	Table S3
Retained: review on the topic	1	Ref. [13]
Excluded: extract used as a synthesis, reducing or exfoliation reagent	6	Table S4
Excluded: proteins, photosystems or intact plant tissue	4	Table S4
Excluded: modifier not of plant origin	14	Table S4
Excluded: other reactions, reviews outside scope, or non-eligible record types	8	Table S4
Excluded: carbonized precursor; no retained molecular component	1	Table S4
Total	60	—

Table S3. The 26 retained research articles, with molecular component, assigned primary interfacial role, and molecular readiness level (MRL; Table 3 of the main text).

#	First author, year	Source	Molecule	Interfacial role	MRL	Search
1	Feng Y. 2017	Chinese Chemical Letters [58]	Chlorophyll a; Cu-chlorophyllin	Light harvester	4	primary
2	Roy K. 2020	ACS Applied Materials and Interfaces [73]	Chlorophyll a	Light harvester	4	primary
3	Huang H. 2025	Separation and Purification Technology [66]	Tannic acid	Coordination scaffold	4	primary
4	Wu Y.-T. 2025	ACS Sustainable Resource Management [60]	Chlorophyll extract (algal biomass)	Light harvester	1	primary
5	Lai Y.-S. 2024	ACS Sustainable Chemistry and Engineering [59]	Leaf extract	Light harvester	1	primary
6	Lv Z. 2022	Science China Materials [49]	Chlorophyll a/b mixture, Cu-substituted	Charge-transfer mediator	3	primary
7	Long X. 2021	ACS Sustainable Chemistry and Engineering [69]	Tannic acid	Coordination scaffold	4	primary
8	Prasad M. 2017	ChemistrySelect [57]	Chlorophyll a	Light harvester	4	primary
9	Agarwal P. 2019	Journal of the Electrochemical Society [36]	β-Carotene	Light harvester	4	primary
10	Shi Y. 2018	ACS Energy Letters [17]	Tannic acid	Coordination scaffold	4	primary
11	Li X. 2024	Applied Catalysis B: Environment and Energy [19]	Caffeic acid	Charge-transfer mediator	4	primary
12	Li Y. 2024	Applied Catalysis B: Environmental [18]	Quercetin	Charge-transfer mediator	4	primary
13	Sun H. 2020	ACS Sustainable Chemistry and Engineering [68]	Tannic acid	Coordination scaffold	4	primary
14	Tian T. 2019	Journal of Catalysis [7]	Tannic acid	Charge-transfer mediator	4	primary
15	Lin P. 2022	Solid State Sciences [70]	Tannic acid	Charge-transfer mediator	4	primary
16	Shiyani T. 2020	Nanomaterials and Energy [24]	Plant extracts	Light harvester	1	primary
17	Hu Y. 2024	Journal of Alloys and Compounds [64]	Tannic acid	Coordination scaffold	4	primary
18	Jaafar S.N.H. 2014	IET Seminar Digest [25]	Pitaya peel extract	Light harvester	1	primary
19	Lai Y.-S. 2022	ACS Agricultural Science and Technology [56]	Leaf extracts	Light harvester	1	primary
20	Huang H. 2024	Journal of Colloid and Interface Science [65]	Tannic acid	Charge-transfer mediator	4	primary
21	Long X. 2025	ACS Applied Energy Materials [16]	Tannic acid	Charge-transfer mediator	4	primary
22	Wang L. 2024	Applied Surface Science [63]	Tannic acid	Coordination scaffold	4	primary
23	Jaafar S.N.H. 2016	Malaysian Journal of Analytical Sciences [26]	Pitaya peel extract	Light harvester	1	primary
24	Ba K. 2025	Separation and Purification Technology [62]	Gallic acid, tannic acid	Coordination scaffold	4	suppl.
25	Huang H. 2025	Chemical Engineering Journal [67]	Ellagic acid	Charge-transfer mediator	4	suppl.
26	Tachan Z. 2014	Advanced Energy Materials [29]	Catechol	Light harvester	4	suppl.

MRLs reflect reported component identity, not verified purity or operating-state integrity: 19 studies are MRL 4, one is MRL 3, and six are MRL 1. No study is assigned MRL 2 or 5; absence of MRL 5 does not establish absence of modified derivatives. Each study is counted once by primary role; Ref. [49] is assigned charge-transfer mediation, consistent with Table 5. Ref. [60] used an uncharacterized algal extract and is assigned MRL 1; its shared chlorophyll scaffold supports inclusion. Ref. [49] used a commercial chlorophyll a/b mixture treated with copper sulfate. The article reports UV-vis, FTIR, XPS, and elemental characterization, but no constituent-resolved LC-MS or NMR quantification in the main article. MRL 3 denotes named mixture constituents under Table 3, not quantitative profiling or complete characterization of the modified derivative.

Primary interfacial roles are descriptive assignments used for counting, not proof of a unique mechanism. Light harvesting is assigned when the study centers on a molecular chromophore or interfacial optical transition. Charge-transfer mediation is assigned when the molecular component is discussed primarily as a bridge or transport layer. Coordination scaffold is assigned when assembly of a metal-containing catalytic overlayer is the principal architecture. Secondary functions may coexist. Reference numbers in Table S3 correspond to the main manuscript.

Table S4. The 33 excluded records and their reasons for exclusion.

#	First author, year	Source	Reason for exclusion
1	Lin G. 2023	ACS Materials Letters	Pigment–protein light-harvesting complex
2	Tian T. 2022	Renewable Energy	Modifier is FeOOH, not of plant origin
3	Fan M. 2023	Advanced Materials Technologies	Modifier is copper phthalocyanine
4	Lal S. 2023	Colloids and Surfaces A: Physicochemical and Engineering Aspects	Leaf extract used as a synthesis-mediating reagent
5	Han Y. 2023	ACS Applied Materials and Interfaces [79]	Target reaction is glycerol valorization
6	Pan J.-B. 2022	Applied Catalysis B: Environmental	Modifier is a metal–organic framework
7	Gagliardi C.J. 2010	Coordination Chemistry Reviews	General review of proton-coupled electron transfer
8	Jin W. 2023	Applied Catalysis B: Environmental	Silicon artificial leaf, no plant molecule
9	Liu S.-S. 2016	Journal of Electrochemistry	Synthetic D–π–A porphyrins
10	Shlosberg Y. 2022	Biosensors and Bioelectronics	Intact plant leaves
11	Tang J. 2022	Journal of Photochemistry and Photobiology A: Chemistry	Caffeic acid-assisted Fe-MOF synthesis; retained molecular function at the operating interface not established in the screened record [71]
12	Huang M. 2020	Nano Energy	Synthetic molecular catalyst
13	Christwardana M. 2022	International Journal of Renewable Energy Development	Bioelectricity generation, not water splitting
14	Jarhad K.V.R. 2025	BioNanoScience [22]	Extract used as a reducing reagent
15	Shiyani T. 2022	IETE Journal of Research	Dye-sensitized solar cell, not water splitting
16	Zhang J.Z. 2016	Nature Chemical Biology	Photosystem II electrode
17	Fernández-Domene R.M. 2024	Journal of Alloys and Compounds [21]	Extract used in anodization synthesis
18	Singh A. 2022	Materials Advances	Application survey, not a PEC study
19	Shoyhet H. 2021	Journal of Materials Chemistry A	Photosystem II hybrid
20	Chen I.-W.P. 2021	Nanomaterials	Chlorophyll used as an exfoliation reagent
21	Lee M.G. 2023	Carbon Energy	Modifier not of plant origin
22	Li J. 2025	Chemical Engineering Journal [30]	Synthetic HHTP-based Co-CAT framework; ligand is not a plant metabolite
23	Huang Z. 2025	International Journal of Hydrogen Energy	Synthetic catechol polymer
24	Jian J. 2022	Advanced Materials	Laser-derived interfacial layers, not of plant origin
25	Huang M. 2020	Journal of Materials Chemistry A	Modifier not of plant origin
26	Lee D. 2020	ACS Applied Materials and Interfaces	Metal–catechol complex consumed in synthesis
27	Jin W. 2020	ACS Sustainable Chemistry and Engineering	Metal recovery, not water splitting
28	Sureshkumar K. 2023	Resource Recovery in Industrial Waste Waters	Metal recovery, not water splitting
29	Zhang Y. 2020	Energy and Environmental Science	Gold nanocatalyst, not of plant origin
30	Berger T. 2013	ChemPhysChem	Surface study, not of plant origin
31	Liu J. 2025	Separation and Purification Technology	Corrigendum; degradation target, not water splitting
32	Liu X. 2022	Chemical Engineering Journal	Polydopamine, not of plant origin
33	Choi Y. 2021	Journal of Materials Chemistry A [28]	Tannic acid consumed during carbonization; carbon layer rather than retained molecular component

Optical upper-bound calculation

For $\varepsilon = 7.6 \times 10^4\ M^{-1}\ cm^{-1}$ and $\Gamma = 0.1\ nmol\ cm^{-2}$, that is $1.0 \times 10^{-10}\ mol\ cm^{-2}$, the absorbance $A = 1000\varepsilon\Gamma$ with Γ expressed in mol cm^{-2} gives 0.0076, and $1 - 10^{-A} = 0.01735$ (approximately 1.7%). The ASTM G173 global-tilt irradiance was converted to photon flux and integrated from 640 to 680 nm using the tabulated wavelength grid. The unit-collection current was 2.9768 mA cm^{-2}. Multiplication by the rounded peak absorbed fraction of 0.017 gives 0.0506 mA cm^{-2}. Applying peak absorption across the interval and assuming complete collection gives an upper bound for that interval only. The Soret band and charge-transfer absorption are excluded. Source: Ref. [61] of the main text.

Table S5. Molecular orbital estimates and electrochemical reference values for Figure 4.

**Component**	**HOMO / LUMO or redox value (V)**	**Source**	**Quantity**
Chl-COOH	+0.34 / −1.42	[40]	Converted orbital energies; not formal redox potentials
Car-COOH	+0.34 / −2.02	[40]	Converted orbital energies; not formal redox potentials
Chl-Car dyad	+0.30 / −1.44	[40]	Converted orbital energies; not formal redox potentials
β-Carotene	+0.47 / −1.91	[39]	Converted orbital energies; not formal redox potentials
Crocetin ester	+0.89 / −1.69	[39]	Converted orbital energies; not formal redox potentials
Synthetic NQ dye	+2.10 / −0.66	[47]	Converted orbital energies; not formal redox potentials
Hydroquinone/benzoquinone	Approximately +0.70 at pH 0	Ref. [44], Table IV	Conventional two-electron reduction potential; not an individual one-electron step
Catechol/o-quinone	Approximately +0.79 at pH 0	Ref. [44], Table IV, note d	Conventional two-electron reference potential; not an individual one-electron step
Betanin	+0.62 at pH 7.4	[42]	Anodic peak; approximately +1.06 V vs. RHE after conversion

Calculated orbital energies were converted using $E = -E(\text{vacuum}) - 4.44$. The resulting NHE-equivalent positions are not measured redox potentials. The conventional two-electron reference values at pH 0 are approximately +0.70 V for hydroquinone/benzoquinone and +0.79 V for catechol/o-quinone [44]. The betanin anodic peak is plotted at +0.62 V vs. NHE at pH 7.4 in Figure 4 [42], equivalent to approximately +1.06 V vs. RHE. NHE and RHE coincide numerically at pH 0; the numerical conversion of orbital energies does not establish a molecular pH response.
